# Expert Clinical Consensus on Body Surface Gastric Mapping Phenotypes for Gastroduodenal Disorders: ‘Auckland Classification’ v1.0

**DOI:** 10.1111/nmo.70377

**Published:** 2026-07-01

**Authors:** Chris Varghese, Nicky Dachs, Gabriel Schamberg, Thomas Abell, Mohammad Al‐Haddad, Timothy Robert Angeli‐Gordon, Shahin Ayazi, Homira Ayubi, Mohammad Bashashati, Robert Bulat, Stefan Calder, Christopher Brian Cederwall, Bianca Chang, Leo Cheng, John Clarke, Charlotte Daker, Peng Du, Jon Erickson, Sven Eriksson, Asma Fikree, Daphne Foong, Mark Fox, Uday Chand Ghoshal, Mahesh Goenka, Rehan Haidry, William L. Hasler, Bu'Hussain Hayee, Vincent Ho, Gerald Holtman, Anthony Robert Hobson, I‐Hsuan Huang, Daniel Keszthelyi, Sahib S. Khalsa, Natasha Koloski, Braden Kuo, David Kunkel, Mikaela Law, David Levinthal, Joy J. Liu, Prateek Mathur, Baharak Moshiree, Leila Neshatian, Catherine Ngo, Linda Nguyen, Nicholas R. Oblizajek, Henry P. Parkman, Sanjay Pandanaboyana, Alexander Podboy, Ali Rezaie, Amol Sharma, Sam Simmonds, Jonathan Sivakumar, Jarongkorn Sirimongkolkasem, Irene Sonu, Abigail Stocker, Joseph Sujka, Jan Tack, Charles Verdonk, Xiao Jing Wang, Douglas Weinstein, John Windsor, Nicha Wongjarupong, William Xu, Natalia Zarate‐Lopez, Christopher N. Andrews, Armen A. Gharibans, Greg O'Grady

**Affiliations:** ^1^ Department of Surgery The University of Auckland Auckland New Zealand; ^2^ Mayo Clinic Rochester Minnesota USA; ^3^ Alimetry Ltd Auckland New Zealand; ^4^ University of Louisville Louisville Kentucky USA; ^5^ Indiana University School of Medicine Indianapolis Indiana USA; ^6^ Auckland Bioengineering Institute, University of Auckland Auckland New Zealand; ^7^ Te Wananga o Aotearoa Auckland New Zealand; ^8^ Allegheny Health Network Pittsburgh Pennsylvania USA; ^9^ King's College Hospital NHS Foundation Trust London UK; ^10^ Dell Medical School, University of Texas at Austin Austin Texas USA; ^11^ Johns Hopkins University Baltimore Maryland USA; ^12^ Wellington Hospital Wellington New Zealand; ^13^ Cedars‐Sinai Los Angeles California USA; ^14^ Stanford University Stanford California USA; ^15^ Waitemata District Health Board Auckland New Zealand; ^16^ Washington and Lee University Lexington Virginia USA; ^17^ University College London London UK; ^18^ School of Medicine, Western Sydney University Sydney New South Wales Australia; ^19^ Klinik Arlesheim and University of Zurich Zurich Switzerland; ^20^ Apollo Multispeciality Hospitals Kolkata West Bengal India; ^21^ Cleveland Clinic London London UK; ^22^ Mayo Clinic Scottsdale Arizona USA; ^23^ Princess Alexandra Hospital; University of Queensland Brisbane Queensland Australia; ^24^ Functional Gut Clinic London UK; ^25^ Tri‐Service General Hospital, National Defense Medical University Taipei Taiwan; ^26^ Maastricht University Medical Center Maastricht the Netherlands; ^27^ Laureate Institute for Brain Research (LIBR), UCLA Los Angeles California USA; ^28^ Vagelos College of Medicine, Columbia University New York New York USA; ^29^ University of California San Diego California USA; ^30^ University of Pittsburgh Medical Center Pittsburgh Pennsylvania USA; ^31^ Feinberg School of Medicine, Northwestern University Chicago Illinois USA; ^32^ Atrium Health, Wake Forest School of Medicine Winston‐Salem North Carolina USA; ^33^ Hoag Digestive Health Institute Newport Beach California USA; ^34^ Temple University School of Medicine Philadelphia Pennsylvania USA; ^35^ Freeman Hospital Newcastle UK; ^36^ Medical University of South Carolina Charleston South Carolina USA; ^37^ University of Melbourne Melbourne Victoria Australia; ^38^ Chulalongkorn University Bangkok Thailand; ^39^ University of South Florida Tampa Florida USA; ^40^ University Hospitals Leuven Leuven Belgium; ^41^ French Armed Forces Biomedical Research Institute Bretigny‐sur‐Orge France; ^42^ Hackensack Meridian Jersey Shore University Medical Center Neptune City New Jersey USA; ^43^ Bangkok Hospital Pattaya Chonburi Thailand; ^44^ Department of Gastroenterology University of Calgary Calgary Alberta Canada

**Keywords:** body surface gastric mapping, chronic nausea and vomiting syndromes, diagnostics, electrogastrography, electrophysiology, functional dyspepsia, gastroparesis, high‐resolution electrogastrography

## Abstract

**Introduction:**

Chronic gastroduodenal disorders remain challenging to manage, and new diagnostic approaches are needed to better delineate underlying causes and guide therapeutic decisions. Body Surface Gastric Mapping (BSGM) technologies combine high‐resolution gastric myoelectrical activity measurements with symptom and psychological profiling to provide mechanistic insights into gastric motor and sensory dysfunction. An International Working Group convened to derive the first consensus classification of BSGM phenotypes (the “Auckland Classification”).

**Methods:**

A Technical Group conducted a systematic literature and clinical database review to identify objective test biomarkers and candidate disease mechanisms. Evidence was synthesized across 50 studies (primarily in gastroparesis, chronic nausea and vomiting, and functional dyspepsia), and BSGM phenotypes were mapped to existing treatment guidelines. Subsequently, iterative review and development of consensus was performed by a Consensus Group composed of international clinical experts familiar with BSGM. Eleven statements underlying the classification were then derived and circulated as a final survey to establish agreement.

**Results:**

Six BSGM phenotypes were endorsed: three defined by myoelectrical abnormalities (Dysrhythmic, High Frequency, and Low Meal Response) and three by characteristic symptom associations (Sensorimotor, Continuous, and Delayed Onset Symptoms). Published studies plausibly linked these phenotypes to mechanisms including interstitial cell of Cajal depletion, vagal impairment, hypomotility, visceral hypersensitivity, gut‐brain dysregulation, and small bowel dysfunction. Phenotypes were also mapped to existing mechanism‐based treatment guidelines. Ten out of the eleven statements had > 80% agreement.

**Conclusions:**

The Auckland Classification, derived by international consensus, presents a structured framework for BSGM‐defined patient phenotypes. Evidence and mechanism‐based treatment options are suggested for each phenotype to provide a foundation for research, further validation, and a pathway for integrating BSGM into clinical care.

## Plain Language Summary

The Auckland Classification v1.0, derived by international consensus, presents a structured framework for body surface gastric mapping‐defined patient phenotypes. This classification provides a foundation for research and a standardized pathway for integrating BSGM into clinical care.

## Graphical abstract

The Auckland Classification, derived by international consensus, presents a structured framework for BSGM‐defined patient phenotypes. This classification forms a foundation for research, further validation, and a pathway for integrating BSGM into clinical care.

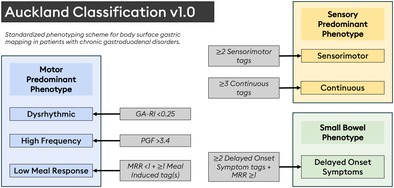

## 1 Introduction

Chronic gastroduodenal symptoms affect around 10% of the global population, with significant impacts to quality of life and healthcare systems [1, 2], yet effective management remains a considerable unmet need [3]. A fundamental challenge has been effectively defining and diagnosing these disorders, with current frameworks presenting overlapping profiles with limited specificity for stratifying patients into clear and effective therapeutic pathways [4, 5, 6].

Gastric emptying studies remain the only widely available test of gastric function [7, 8, 9]. Although gastric emptying testing is considered a diagnostic standard in gastroparesis, the tests lack specificity for underlying disease mechanisms because several distinct physiological factors regulate emptying [10, 11, 12]. This heterogeneity contributes to variable test–retest reliability and mixed results when predicting treatment response and selecting treatment options [4, 5, 6, 9, 13]. Tests assessing disease mechanisms beyond gastric emptying alone might offer more specific and effective therapeutic targets [4, 12, 14].

**FIGURE 1:**
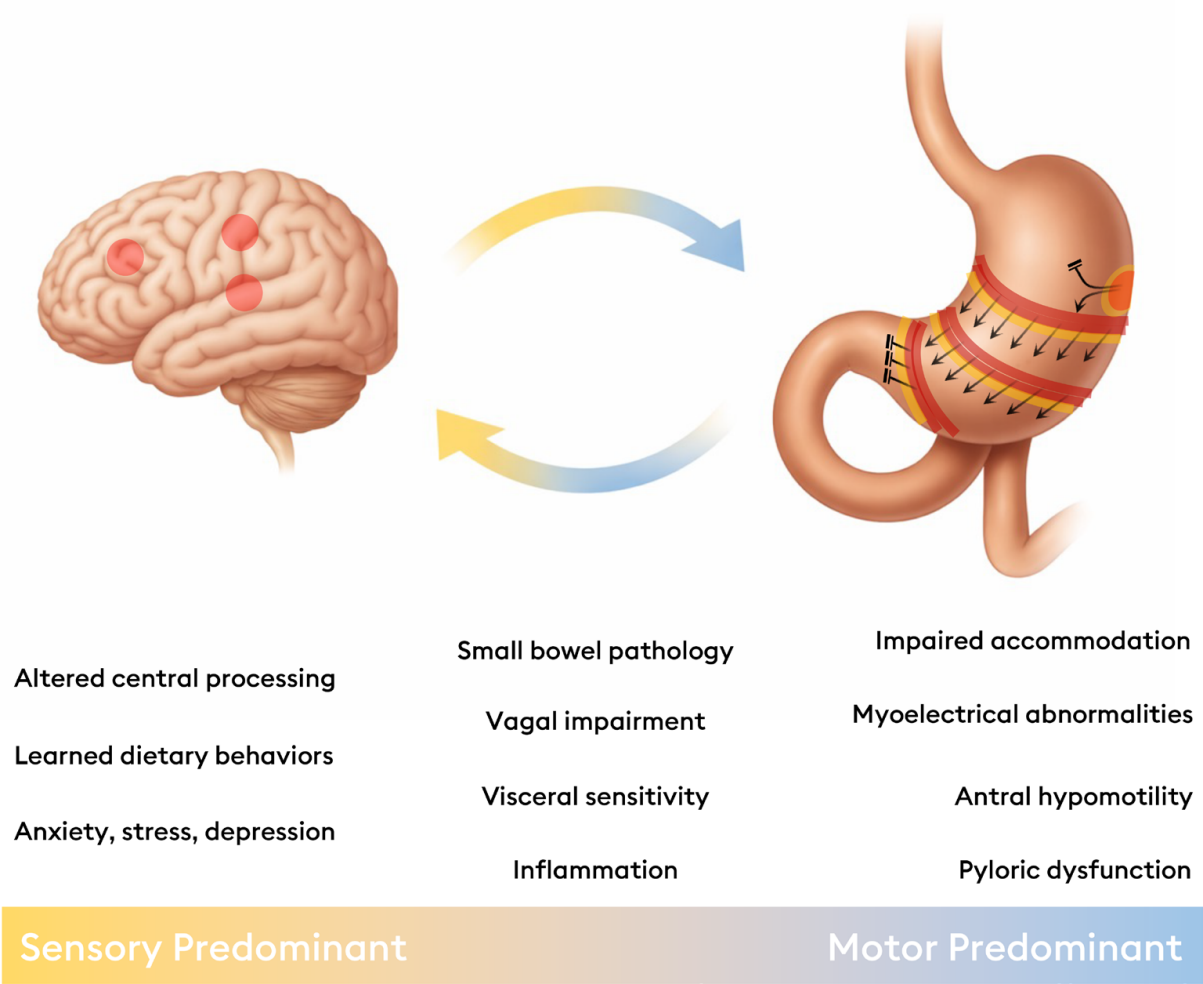
Conceptual framework stratifying gastroduodenal symptom drivers along a spectrum from sensory predominant to motor predominant, in conjunction with bidirectional gut–brain axis links. BSGM methods aid in characterizing individual factors within this framework from diverse pathophysiologies, including myoelectrical abnormalities, gastric dysmotility, visceral sensitivity, vagal impairment, altered central processing, and psychological factors (anxiety, stress, and depression). Assessing transit, accommodation, pyloric function, and small bowel factors requires complementary diagnostic modalities.

Additional tests of gastroduodenal function include antroduodenal manometry (ADM), gastric accommodation testing (e.g., barostat, single photon emission computed tomography, nutrient drink, or satiety tests), and low‐resolution electrogastrography (EGG) [8, 15, 16]. These tests have provided meaningful insights into mechanisms underlying gastroduodenal dysfunction and the causes of dyspeptic symptoms after meals (e.g., Figure 1). However, in clinical practice they may be limited by low availability, invasiveness, uncomfortable data acquisition, complex interpretation, or inconsistent utility [7, 17]. Consequently, care decisions continue to be driven by symptom patterns and trial‐and‐error management [18, 19].

The need for more accurate and mechanistic diagnoses motivated the development and clinical translation of a new non‐invasive diagnostic test for assessing mechanisms underlying chronic gastroduodenal symptoms [20, 21, 22]. This platform, Body Surface Gastric Mapping (BSGM), was built upon technical advances in high‐resolution electrophysiology, superseding low‐resolution EGG [23, 24, 25]. Research grade devices have matured, with an FDA‐cleared clinical system first released in 2023 (Gastric Alimetry, New Zealand), and recently approved in pediatrics aged ≥ 12 years. This system combines BSGM with digital symptom and psychological profiling in order to offer a comprehensive, non‐invasive, and radiation‐free diagnostic approach [20, 22, 26, 27]. There are now more than 50 clinical studies reported in the literature.

The International Body Surface Gastric Mapping Working Group (herein “Working Group”) previously convened to conduct a technical review and establish an evidence‐based consensus on the principles, technical foundations, and clinical methods of BSGM [28], and has also provided report interpretation guidelines [29]. The Working Group has now proceeded to review the available clinical data, propose a consensus “Auckland Classification” for chronic gastroduodenal disorders based on BSGM, and map the phenotypes to potential therapeutic options. These developments are intended to standardize terminology, guide future research, and inform clinical implementation of BSGM.

## 2 Methods

The Working Group is composed of gastroenterologists, surgeons, physiologists, psychiatrists, scientists, and bioengineers with relevant research and/or clinical experience, who convened to define an inaugural classification scheme for BSGM. The Working Group comprised two groups: first, a “Technical Group” comprising four expert clinicians, five clinical researchers and bioengineering specialists, and one health psychologist, who performed the systematic review and collated a draft proposal; and second, a “Consensus Group,” comprised of more than 45 clinical and research experts familiar with BSGM (ranging from routine clinical use to familiarity with technology without previous research or clinical experience; the latter group being invited for their clinical expertise), who provided iterative review, feedback, and consensus. The “Consensus Group” was selected based on established research experience with BSGM technology and/or relevant clinical expertise in gastroduodenal disorders. Selection was aimed at incorporating geographical representation and including participants from diverse clinical practice and research settings.

### 2.1 Search Strategy

A comprehensive literature search using Ovid Medline and EMBASE databases from inception to August 2025 was performed. The first search included the terms “body surface gastric mapping” OR “gastric mapping” OR “BSGM” OR “Gastric Alimetry” OR “high‐resolution electrogastrography” OR “HR‐EGG,” and clinical and translational studies were retrieved. A second search then used society names to capture therapeutic guidelines. The latest guidelines published by the American Neurogastroenterology and Motility Society (ANMS), European Society of Neurogastroenterology and Motility (ESNM), American College of Gastroenterology (ACG), American Gastroenterological Association (AGA), British Society of Gastroenterology (BSG), and United European Gastroenterology (UEG) for “gastroparesis,” “chronic nausea and vomiting syndromes,” “functional dyspepsia,” and “gastric sensorimotor” disorders were additionally retrieved. BSGM biomarkers and BSGM phenotypes were defined and subsequently mapped to therapies from guidelines. Article bibliographies were screened to ensure comprehensive literature capture.

Studies on low‐resolution EGG techniques and associated wearable motility patches were excluded, as the Working Group agreed that low‐resolution techniques are not clinically equivalent to BSGM and do not represent comparable services from a technical, clinical utility, resource requirement, or reimbursement viewpoint. The justification was that a minimum of 32 simultaneous gastric electrodes are currently deemed necessary to class as high‐resolution recordings [28]. Clinical data arising from single‐channel electrophysiology were therefore not considered relevant to inform the phenotyping classification, although low‐resolution EGG studies provided valuable insights into foundational pathophysiology.

### 2.2 Evidence Synthesis

Based on the search results, a narrative synthesis of the evidence as relevant to the proposed phenotyping scheme was integrated into an initial working document by the Technical Group. The study cohort, complementary diagnostic modality, and treatment being investigated in each study are tabulated and referenced to support each phenotype, their proposed diagnostic criteria, putative pathophysiology, and therapeutic research consideration. This synthesis detailed the evidence base for each BSGM phenotype and informed the synthesis of the new classification system. Guided by other classification schemes for complex GI disorders, the Group aimed to prioritize a hierarchy of: (i) organic disorders/structural pathology; (ii) motor function; and (iii) abnormal sensitivity [30]. In addition, plausible (research‐informing) therapeutic considerations anchored to existing guidelines were identified for future dedicated studies. Priority research gaps and directions for future work were identified. It is noted that this must not be considered as a therapeutic guideline. An initial document was then circulated to all members of the Consensus Group for review, comment, and iterative refinement across two rounds. Expert consensus was used to formalize and standardize phenotype definitions and nomenclature, potential contributory mechanisms (summarized into a conceptual diagram in Figure 1), suggested mechanism‐targeted therapies for further investigation, and future research prioritisation. A separate pediatric classification scheme is presently under development, with adolescent reference intervals now available [31].

### 2.3 Phenotype Prevalence

An estimation of the prevalence was presented for each phenotype, based on a multicenter database of patients that have undergone BSGM and formal gastric emptying testing (further detailed in Appendix E).

### 2.4 Consensus Voting and Agreement

A modified Delphi approach, similar to what has been used in previous GI consensus statements was employed [32, 33]. Literature and expert inputs formed the basis of this document, reflecting a v1.0 classification. In this process, the Technical Group shortlisted the initial phenotypes with corresponding proposed diagnostic criteria, putative pathophysiology's and clinical implications for each phenotype. This text was iteratively refined across 2 rounds of feedback from the “Consensus Group.” Thereafter, the Technical Group distilled the refined document into 11 summary statements which all members of the Consensus Group were invited to vote on using a standard 6‐point scale for assessment of agreement level: A+, agree strongly; A, agree with minor reservation; A−, agree with major reservation; D−, disagree with major reservation; D, disagree with minor reservation; D+, disagree strongly [32]. The members were instructed to evaluate the phenotype definitions, their clinical significance, and associated criteria based on appropriateness for a Version 1.0 framework. They were asked to specifically evaluate phenotype criteria on their appropriateness as a common language for standardized reporting, not as strict thresholds that supersede clinical judgment in the phenotyping process. Per‐statement agreement level was defined as the percentage of responses rated either A+ or A. The statements, agreement level, and percentage of each rating are provided in Table 1.

**TABLE 1:** Auckland Classification summary and level of agreement.

Phenotype category	Statement	Criteria	Level of agreement
Motor predominant phenotypes	The “Dysrhythmic” phenotype is defined by sustained dysrhythmia and is indicative of gastric neuromuscular dysfunction	Primary criterion: Overall Gastric Alimetry Rhythm Index (GA‐RI) < 0.22. Secondary criterion: Overall GA‐RI < 0.25	47 responses with 93.6% agreement (61.7% A+, 31.9% A, 6.4% A‐, 0.0% D−, 0.0% D, 0.0% D+)
	A severely abnormal rhythm (overall GA‐RI < 0.22) should be prioritized as the highest‐order finding, superseding all other motor or sensory phenotypes	Overall GA‐RI < 0.22	47 responses with 85.1% agreement (51.1% A+, 34.0% A, 10.6% A−, 2.1% D−, 2.1% D, 0.0% D+)
	The “High Frequency” phenotype is defined by a stable high Principal Gastric Frequency (PGF) and can be associated with vagal nerve dysfunction	Primary criterion: Overall PGF > 3.4 cpm. Secondary criterion: Overall PGF > 3.35 cpm	47 responses with 83.0% agreement (48.9% A+, 34.0% A, 17.0% A−, 0.0% D−, 0.0% D, 0.0% D+)
	The “Low Meal Response” phenotype is defined by low and/or delayed gastric activity in response to a meal with meal‐induced symptoms and is indicative of a relatively weak or delayed reaction soon after the meal	Primary criteria: Meal Response Ratio (MRR) < 1 and 1 or more symptoms with meal induced tags. Secondary criteria: BMI‐Adjusted amplitude < 22 μV overall or in either of the first two postprandial hours and 1 or more symptoms with meal induced tags	47 responses with 80.9% agreement (44.7% A+, 36.2% A, 19.1% A−, 0.0% D−, 0.0% D, 0.0% D+)
Sensory predominant and small bowel phenotypes	The “Sensorimotor” phenotype is defined by correlation between symptoms and gastric amplitude, implying that symptoms are associated with gastric motility, and can be associated with gastric hypersensitivity	Primary criterion: 2 or more symptoms with sensorimotor tags (correlation > 0.5). Secondary criterion: 1 or more symptoms with sensorimotor tags	47 responses with 85.1% agreement (42.6% A+, 42.6% A, 10.6% A−, 4.3% D−, 0.0% D, 0.0% D+)
	The “Continuous” phenotype is defined by persistent and stable symptom severity and implies that gastric activity does not contribute to changes in symptoms	Primary criterion: 3 or more symptoms with continuous tags. Secondary criterion: 1 or 2 symptoms with continuous tags	47 responses with 97.9% agreement (53.2% A+, 44.7% A, 0.0% A−, 2.1% D−, 0.0% D, 0.0% D+)
	The “Delayed Onset Symptoms” phenotype is defined by symptoms peaking in the late postprandial period, after the majority of gastric activity, and indicates that symptoms likely arise distal to the stomach	Primary criteria: 2 or more symptoms with late onset tags and MRR ≥ 1. Secondary criterion: 1 or more symptoms with late onset tags	47 responses with 78.7% agreement (46.8% A+, 31.9% A, 19.1% A−, 2.1% D−, 0.0% D, 0.0% D+)
Emerging phenotypes	Low frequency gastric activity is categorized as an “emerging” phenotype and is defined by a low Principal Gastric Frequency (overall PGF < 2.65 cpm). This is an important area for future research and evaluation, but clinical utility and evidence are not yet sufficient to include this as a core phenotype in version 1.0	Overall PGF < 2.65 cpm	47 responses with 87.2% agreement (55.3% A+, 31.9% A, 10.6% A−, 2.1% D−, 0.0% D, 0.0% D+)
	High amplitude gastric activity is categorized as an “emerging” phenotype and is defined by a high BMI‐Adjusted Amplitude (overall BMI‐Adjusted Amplitude > 70 μV). This is an important area for future research and evaluation, but clinical utility and evidence are not yet sufficient to include this as a core phenotype in version 1.0	Overall BMI‐Adjusted Amplitude > 70 μV	47 responses with 83.0% agreement (44.7% A+, 38.3% A, 14.9% A−, 0.0% D−, 2.1% D, 0.0% D+)
	In the absence of evidence supporting the 6 core phenotypes, it is appropriate to assess other areas, such as spectral metrics outside the normative range, symptom severity and relation to a meal, and mental wellbeing	Not applicable	47 responses with 93.6% agreement (76.6% A+, 17.0% A, 6.4% A−, 0.0% D−, 0.0% D, 0.0% D+)
General consensus	Taking the proposed classification in its entirety and given the emerging evidence in this field, it is appropriate to adopt this classification as the initial version of a common framework for standardized clinical reporting and future research	Not applicable	47 responses with 97.9% agreement (76.6% A+, 21.3% A, 0.0% A−, 0.0% D−, 0.0% D, 2.1% D+)

## 3 BSGM Methods

The evidence synthesis presented below briefly covers the standardized principles, protocols, and metrics for BSGM testing, before presentation of the “Auckland Classification” phenotype consensus.

### 3.1 Principles and Protocols of BSGM Testing

BSGM is indicated in patients with chronic gastroduodenal symptoms in order to determine specific motor and sensory mechanisms contributing to symptoms, such as myoelectrical dysfunction, hypomotility, visceral hypersensitivity, and brain–gut axis contributions [11, 20, 34, 35, 36]. The test is used alongside clinical evaluation and other diagnostic tests as an aid to diagnosis.

The principles and clinical methods of BSGM testing were recently summarized in a separate technical review by the Working Group [28]. In brief, BSGM summates all underlying gastric myoelectrical activity, including both propagating slow wave potentials generated by the interstitial cells of Cajal (ICC), as well as associated smooth muscle potentials [22, 37, 38]. Cutaneous gastric signals are weak, being ~100× lower in amplitude than cardiac potentials [22]. Multiple limitations affecting low‐resolution EGG were therefore resolved to enable BSGM, with a particular focus on improving signal‐to‐noise ratio, artifact handling, signal loss due to variable gastric anatomy, and clinical uility [22, 28, 39, 40, 41]. The central innovation was to employ high‐resolution arrays of electrodes covering a wide area to maximally capture gastric activity [22]. These advances have been robustly validated against direct serosal recordings, demonstrating high fidelity [25]. As discussed below, in addition to the acquisition of objective gastric myoelectrical activity, BSGM testing also routinely encompasses detailed subjective symptom profiling before and after ingestion of a standardized nutrient challenge, and an optional psychological screening questionnaire, with these results now being critical adjuncts to phenotyping.

### 3.2 Test Protocol

**FIGURE 2:**
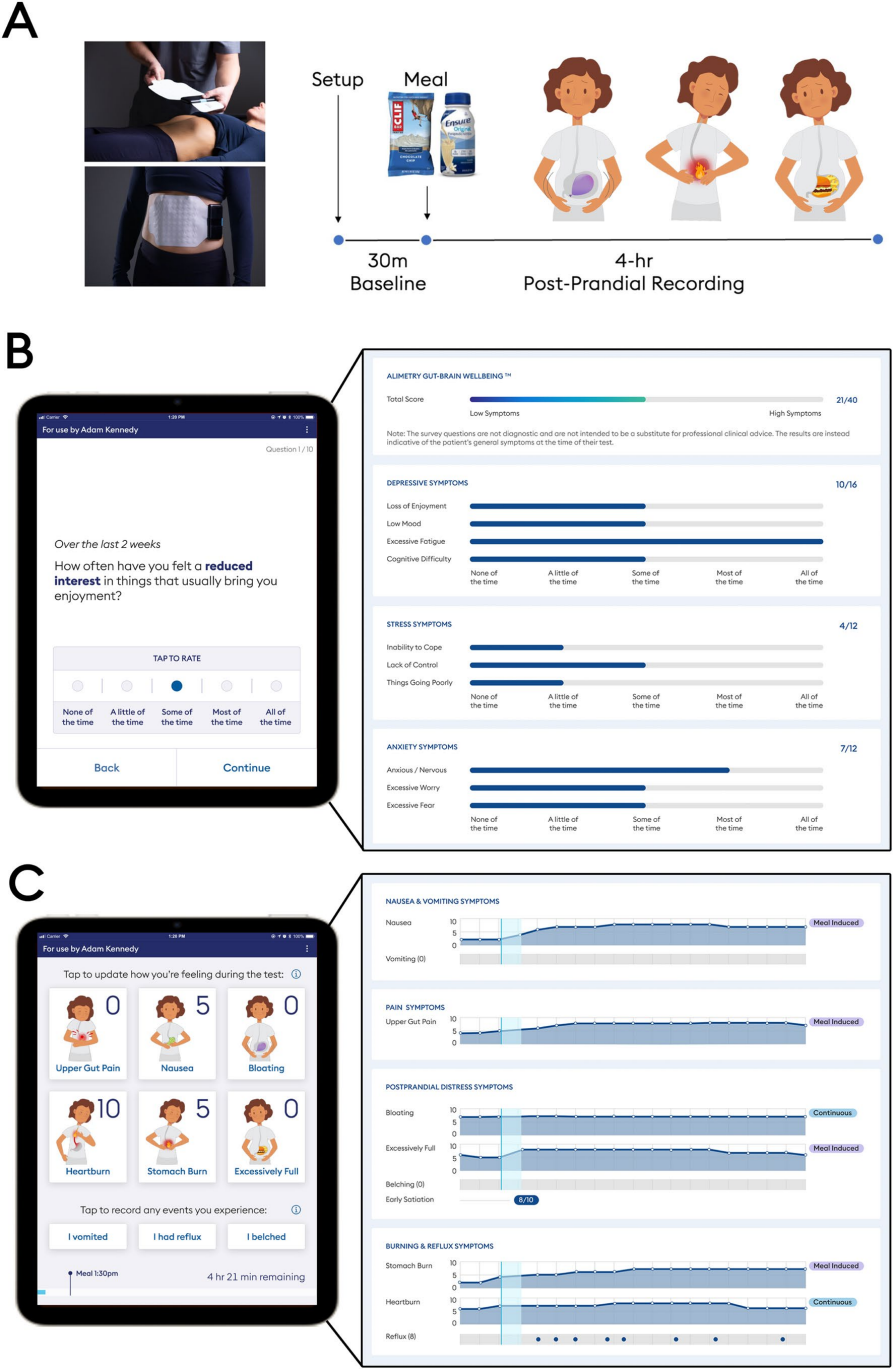
(A) The standard Gastric Alimetry test protocol is 4.5 h, comprising a 30‐min baseline, test meal, and 4‐h post‐prandial recording [22]. (B) Psychometric profiling is optionally performed at the start of the test using the validated Alimetry Gut–Brain Wellbeing Survey [27]. (C) Symptoms are captured at the start of the test and at least every 15 min in a validated App, using a patient dashboard with symptom logging screens [26].

A standardized clinical BSGM testing protocol has been implemented per Figure 2 [28]. This protocol includes a complete gastric meal cycle time of 4 h [20, 22], which is currently necessary to apply normative ranges and phenotyping schemes [34, 42], although shorter protocols are still being investigated, including in mental health research contexts [43]. The commercial Gastric Alimetry System has regulatory clearance for use in patients aged ≥ 12 years old [44]. The test is validated for a patient body mass index (BMI) of up to 35 kg/m^2^, and whilst the test has been anecdotally observed to be effective above this cutoff in selected cases, caution should be exercised when applying the proposed classification scheme to high BMI patients (see Appendix A) [40]. There is no cut‐off for low BMI.

BSGM performed through the Gastric Alimetry test also includes two complementary diagnostic adjuncts delivered in a companion App [26, 27]. Firstly, a symptom profiling App, validated in a study of 79 patients, records typical foregut symptoms at minimum 15‐min intervals (e.g., nausea, pain, bloating, heartburn, stomach burn, upper gut pain, excessive fullness), as one‐off measures (early satiation after meal ingestion), or as discrete time‐stamped events (e.g., vomiting, belching, reflux), with validated pictograms to enhance the reliability of patient self‐reporting [26, 45, 46]. Clinically correlating the resultant symptom curves and events to meal ingestion and gastric meal responses is integral to test interpretation [29, 47]. Secondly, a validated mental health survey is optionally provided in the Gastric Alimetry test (the “Alimetry Gut Brain Wellbeing Survey”; AGBW) [27], encompassing depression, anxiety and stress domains. This is included because psychological comorbidities are common, may reflect shared disease mechanisms, and frequently track with disorder severity [2, 48], while also correlating with specific phenotypes (discussed below) [20, 34, 49]. Due to the multifactorial nature of GI symptoms (Figure 1), the Working Group recommends that these tools should be routinely employed alongside BSGM testing if phenotyping is to be applied.

A standardized meal is given during BSGM testing to provoke a gastric meal response and symptoms, typically being a nutrient/protein bar with nutrient drink, with variants to accommodate dietary requirements or research protocols [28]. The recommended standard meal is consumed over 10 min, and is ~450 kCal (in comparison to gastric emptying test protocols of 255–320 kCal) [13]. The large meal size is motivated by the need to provide a sufficient caloric load to stress gastric motor function and provoke symptoms, offering a more sensitive physiological challenge than lower‐calorie standards, although > 50% meal completion is presently considered sufficient for routine test interpretation [50]. Tests involving smaller meal portions or even fasting tests may still be interpretable; however, low gastric amplitudes and rhythm stability must then be interpreted with caution [51]. Previous MRI studies have indicated that a minimum meal volume (e.g., > 200 mL) is likely required to elicit a reliable physiological response [52]. In addition, researchers have tested various meal compositions (e.g., egg meals, pancake, nutrient bars with water, liquid test meals), concluding that calorie load, as opposed to macronutrient content, appears to be the key determinant in meal response metrics, such that patients may choose to bring their own meal if necessitated by dietary restrictions or rigid preferences [50].

### 3.3 Gastric Alimetry Test Biomarkers

**FIGURE 3:**
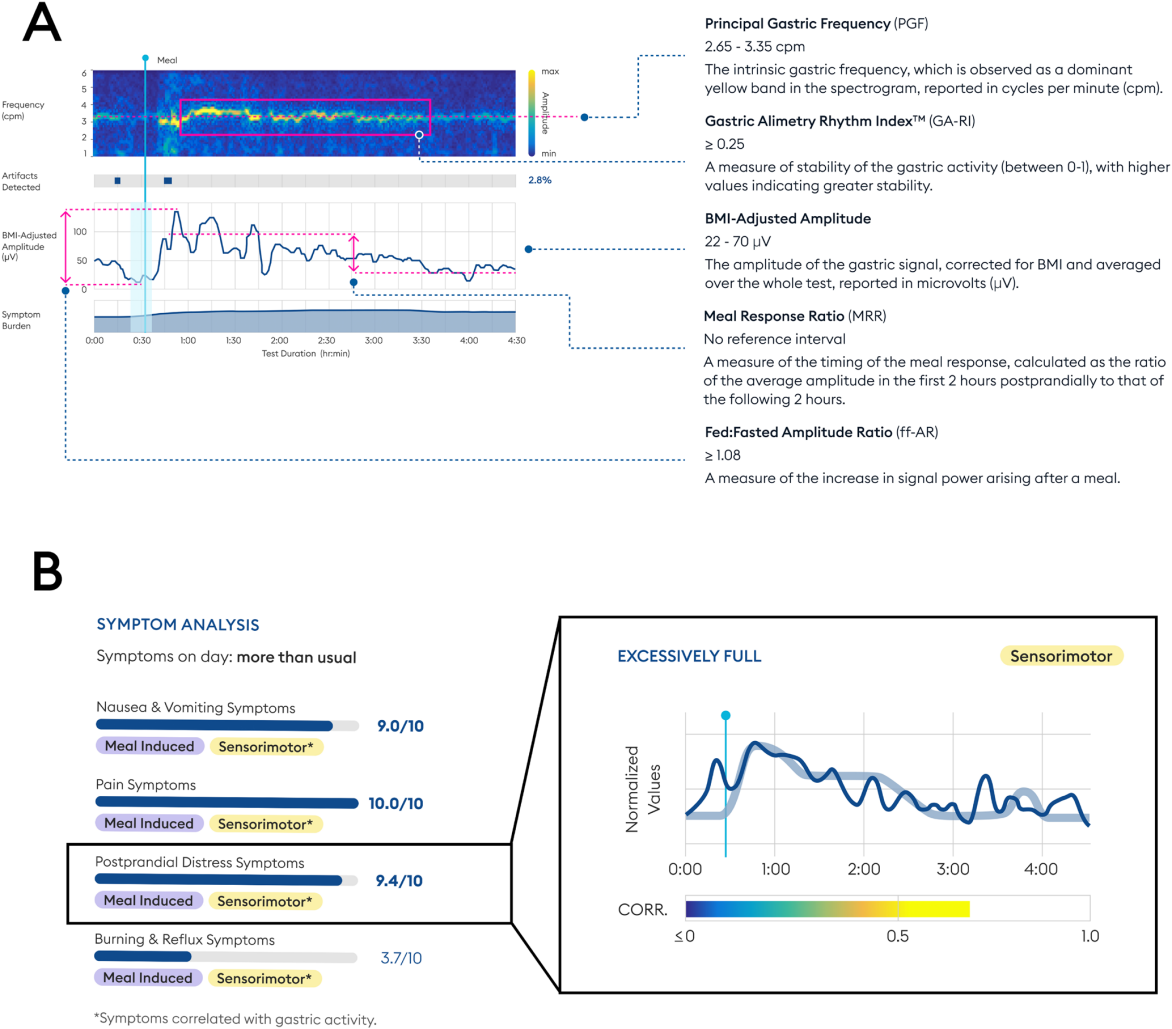
(A) Example frequency‐amplitude spectrogram shown together with normative reference intervals for Gastric Alimetry, derived from a prospective cohort of demographically diverse healthy adults (*n* = 110) [42]. The four spectral metrics are defined with reference to a standardized 4.5‐h test protocol: Gastric Alimetry Rhythm Index (GA‐RI), Principal Gastric Frequency, Fed:Fasted Amplitude Ratio and BMI‐Adjusted Amplitude. Adolescent normative reference intervals have also been published [31]. (B) Example of correlations between gastric amplitude and gastroduodenal symptoms (pain referring specifically to epigastric pain), with a representative example of a high correlation between feelings of excessive fullness and the gastric amplitude curve (correlation strength > 0.5).

BSGM utilizes new metrics that overcome the limitations inherent in low‐resolution EGG biomarkers, including susceptibility to artifacts, conflation of frequency with rhythm stability, and lack of correction for BMI [40, 53]. The three key metrics derived from BSGM are: Principal Gastric Frequency (PGF; reference interval: 2.65–3.35 cpm), Gastric Alimetry Rhythm Index (GA‐RI; ≥ 0.25), and BMI‐adjusted amplitude (22–70 μV) (example spectrogram summarized in Figure 3A) [29, 42]. Reference intervals for these metrics were established in a study of 110 healthy controls of diverse age and ethnicity [29, 42], with strong test–retest reproducibility shown at short (1 week) and long‐term (> 6 month) intervals [54], and resilience to vomiting should it occur during testing [55].

A fourth metric is reported, termed the Fed:Fasted Amplitude Ratio (ff‐AR). However, the Working Group did not find any supporting evidence for the diagnostic utility of ff‐AR at this time. They noted inherently high variability in control subjects and recommended that this metric be reserved for use in research applications or as a supporting metric in otherwise abnormal tests [42]. A fifth exploratory metric used in research studies, called the Meal Response Ratio (MRR), was recommended for ongoing evaluation as a potential replacement for ff‐AR. This additional metric indicates the timing of the meal response and is calculated as the ratio of the average amplitude in the first 2 h postprandially to that of the next 2 h, with ratios < 1 found to be associated with delayed gastric emptying [12]. The core metrics are explained further in Appendix B.

The association between gastric amplitude and symptoms over the test duration is also quantified by a *correlation score* between 0 and 1, which aims to offer a proxy indicator of “sensorimotor disorders” (recommended cut‐off > 0.5) that is, principally gastric hypersensitivity (Figure 3B) [34]. Some Working Group members queried whether disordered accommodation might also express a correlation of > 0.5; however, no data was found in the literature review to support or refute this concept, indicating an area for future research.

Finally, a series of “symptom tags” recently received FDA clearance to objectively profile and categorize individual symptom severity curves reported during testing. These tags include “meal induced,” “meal alleviated,” “late onset,” “sensorimotor” (*correlation score* > 0.5), and “continuous,” as recently described in detail elsewhere [56]. Late‐onset symptoms peak in the late post‐prandial period (hours 3+), a time typically associated with the transition of chyme distal to the stomach, and have therefore been considered a plausible indicator of symptoms of small bowel origin [34, 57].

## 4 Results

### 4.1 BSGM Phenotypes and the Auckland Classification v1.0

Following BSGM metric development, the Working Group focused on identifying a set of well‐characterized “phenotypes” that can underpin disease classifications and clinical decision making [34, 58]. This was achieved by mapping phenotypes to symptom and psychological profiles and to pathophysiological mechanisms (Figure 1) [29, 34, 49, 58].

Here, we present this consensus classification scheme iteratively developed by the Working Group [28, 58], termed the Auckland Classification v1.0, based on current accrued evidence from 50 published studies identified in the literature review, and accumulated research and clinical experience among the Working Group from over 4500 tests (refer to Appendix C, Table S1 for evaluated studies). The phenotypes presented here should be considered the first consensus classification scheme for BSGM, superseding all earlier interim schemes [28, 34], and they are intended to be used alongside other diagnostic tests of gastric function such as emptying testing, if desired. In addition, a deterministic algorithm to objectively ascertain the Auckland Classification from individual BSGM tests was developed by the Technical Group (presented in Appendix D). This could be integrated into automated reporting tools in future versions of BSGM software.

**FIGURE 4:**
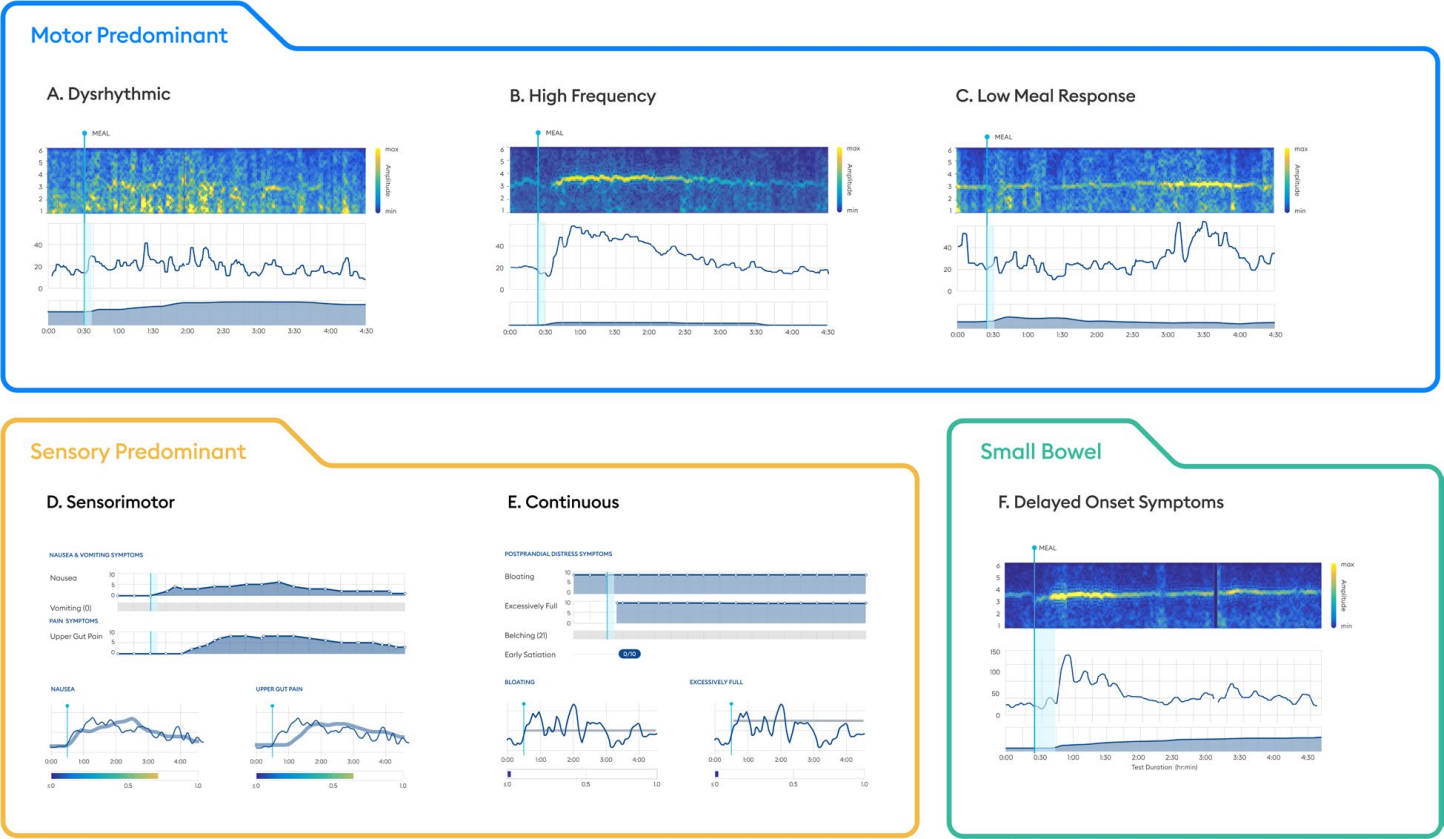
Example spectrograms and symptom profiles of BSGM phenotypes.

**FIGURE 5:**
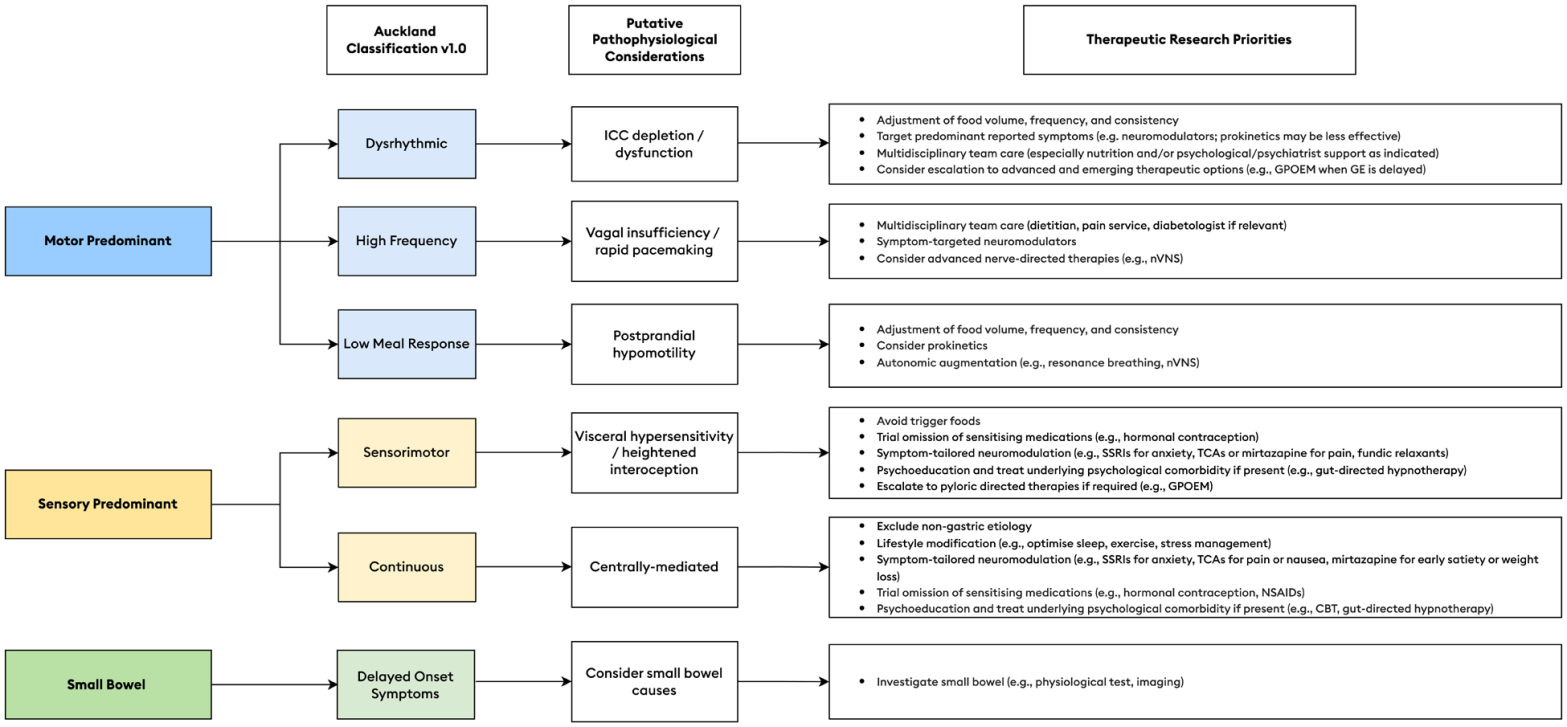
Potential therapeutics research priorities based on Auckland Classification v1.0 phenotypes. The following mechanism‐targeted treatment considerations are based on synthesis of existing guidelines for treatment of gastroparesis and Rome‐IV defined chronic nausea and vomiting syndromes, mapped onto putative pathophysiologies within the Auckland Classification v1.0, as phenotyped using the Gastric Alimetry System. The relationship between phenotype, putative pathophysiology, and their relationship to treatments is under active investigation and should be considered priorities for further research. This diagram should not be considered a therapeutic guideline.

Six phenotypes were resolved by the Working Group and grouped by motor or sensory predominance. These phenotypes are described in detail below along with currently identifiable clinical considerations and research gaps. The phenotypes are illustrated using representative case examples in Figure 4. The consensus statements describing each phenotype, quantitative criteria used in the automated classification algorithm, and associated levels of agreement are reported in Table 1. Acknowledging the difficulty in assigning a strict threshold for classification, we establish two sets of criteria that correspond to the strength of the evidence for each phenotype. Meeting the “Primary Criteria” for a phenotype suggests stronger alignment with that phenotype than only meeting the “Secondary Criteria.” These sets of criteria are used to prioritize phenotypes in the automated classification algorithm (see Table S2), which was subsequently applied to a cohort of 176 patients to estimate the prevalence of each phenotype (Appendix E). Additional emerging phenotypes that did not currently reach sufficient agreement for inclusion in the v1.0 Classification, but signal important ongoing research areas, are also addressed. Lastly, a summary diagram offering research‐oriented treatment considerations for each phenotype is presented in Figure 5.

### 4.2 Auckland Classification: Motor Predominant Phenotypes

#### 4.2.1 Dysrhythmic Phenotype

##### 4.2.1.1 Criteria

Sustained dysrhythmia as indicated by low GA‐RI values (refer to Table 1; e.g., Figure 4A).

##### 4.2.1.2 Pathophysiological Considerations

Disruptions in gastric myoelectrical activity and/or ICC network integrity are now established to play a role in the pathophysiology of several gastroduodenal disorders [4, 59, 60, 61, 62, 63]. Multiple studies, including a recent meta‐analysis, confirmed that ICC network depletion, commonly in association with a decline in anti‐inflammatory macrophages, is a pathological hallmark of severe gastroduodenal disorders, regardless of gastric emptying status [4, 60, 61, 62, 64, 65, 66]. This “Cajal‐opathy” represents a distinct neuromuscular disorder that is not accounted for in current disease classifications [67, 68]. The clinical relevance of this condition is supported by the finding that more severe ICC depletion correlates with higher rates of delayed gastric emptying, although patients with ICC depletion can be found anywhere on a disease spectrum across functional dyspepsia/chronic nausea and vomiting syndromes and gastroparesis [4].

A growing body of evidence indicates that the Dysrhythmic Phenotype, defined by sustained dysrhythmia with GA‐RI < 0.25, offers a plausible diagnostic indicator for ICC depletion with neuromuscular dysfunction [20, 34, 63]. While this linkage is logical, based on evidence from translational studies using invasive serosal mapping in which degraded ICC networks and dysrhythmias were observed [61, 62, 69], direct evidence currently rests on limited data using single‐channel EGG methods, which showed that patients with ICC depletion displayed significantly reduced normal slow wave activity and increased tachygastria in both fasting and fed states compared to those with intact networks [63]. Additional studies employing BSGM are therefore recommended, while noting that the traditional acquisition of full thickness biopsies from the stomach is challenging and invasive. Gastric emptying may be preserved or delayed, depending on whether sufficient electromechanical coupling occurs to facilitate effective bolus propagation [62]. Symptom drivers in this phenotype are likely to include abnormal motor function, together with concomitant immune activation mechanisms [64, 70]. In a recent cohort of 210 patients with chronic gastroduodenal symptoms, 19% of patients showed this phenotype [34, 71]. Rates of this phenotype tend to vary between 10% and 30% depending on referral population (Table S3). Symptom profiles varied, but were most commonly meal‐induced in over two thirds.

The literature review also identified advanced systemic sclerosis as a potential cause of sustained gastric dysrhythmia, potentially related to fibrosis [72]. Numerous other causes of gastric dysrhythmia have been described in the literature, including motion sickness, short‐acting opiates, excessive gastric volume loading, vasopressin, and glucagon [38, 73]. These factors are typically avoided during BSGM testing. In addition, transient dysrhythmias have been observed, typically in the early post‐prandial period, and often correlated to post‐prandial distress [29]. This phenomenon is currently poorly understood and requires further research regarding mechanisms and implications [29].

##### 4.2.1.3 Therapeutic Research Directions

The Dysrhythmic Phenotype has been associated with comorbidities including diabetes and neuropathic diagnoses, plausibly related to pathophysiologic mechanisms discussed above [35]. Poor glycemic control is known to be a potential trigger for ICC depletion [74]. This may be reversible with tightening of glycemic control, which should therefore be prioritized in affected diabetic patients to recover ICC network function [74, 75, 76], however it remains to be determined whether BSGM findings revert to normal with ICC restoration. In addition, Sadaka et al. showed in adolescents that the Dysrhythmic Phenotype was completely correlated with a neuropathic diagnosis by ADM in a cohort of 15 patients, with further validation studies now awaited [77].

The Dysrhythmic Phenotype has also been associated with greater severity of symptoms, particularly in children. Humphrey et al. demonstrated worse symptoms in adolescents with the Dysrhythmic Phenotype compared to all other patients, including more severe upper gut pain and nausea, as well as higher disability scores and reduced quality of life [44]. Sadaka et al. similarly found more severe symptoms of nausea and bloating compared to all other patients in their adolescent study [77]. An adult study of 43 patients with chronic nausea and vomiting and matched controls showed that symptoms correlated with the Dysrhythmic Phenotype when present; whereas symptoms correlated with mental health parameters in patients with normal spectral metrics [20]. Similar findings have since been shown in successive cohorts of increasing size, demonstrating the potential of phenotyping to plausibly distinguish subgroups by disease mechanisms [34, 49]. Overall, the Dysrhythmic Phenotype is therefore currently viewed as indicative of a relatively severe group of patients with true gastric neuromuscular disorders, potentially requiring a higher intensity of medical management [20, 61, 62, 63].

In the absence of disease‐modifying therapies, therapy for the Dysrhythmic Phenotype is currently focused on optimizing gastric motor function, symptom control, and nutritional support. ICC depletion may be expected to impair antral contractility and antro‐pyloric‐duodenal coordination, which may impact the breakdown of solid food (trituration) and gastric emptying [78, 79]. Dietary modification in favor of low volume, small particle size diets is currently recommended to support gastroduodenal transit and alleviate antral distension [80, 81]. However, the Working Group notes that phenotype‐guided dietary studies are currently lacking, indicating a priority area for future research [82, 83]. While symptom management may include antiemetics, emerging evidence suggests that prokinetics may be disadvantageous in this cohort [84], with additional validation pending in a prospective study (NCT06854120). Neuromodulators are also used in patients with gastroduodenal symptom disorders, especially those without gastric emptying delay [85, 86, 87], which can be further informed by mental health overlays given that this phenotype is known to be predisposed to depression and stress associations [88].

As noted above, the Dysrhythmic Phenotype may require treatment escalation owing to the underlying neuromuscular dysfunction, such that comprehensive multidisciplinary input with dietitians, nutritional support teams, and psychological/psychiatric support may be necessary [71, 88, 89]. Currently, the specific prevalence of advanced nutritional support requirements and success rates (e.g., nasogastric, nasojejunal, or parenteral nutrition) in this phenotype is unknown, presenting a valuable area for future research to guide prognostic counseling and proactive management. Data is limited, but in treatment refractory settings where gastric emptying is delayed, this group may be carefully considered, with shared clinician‐patient decision making, for gastric per oral endoscopic pyloromyotomy (GPOEM), in view of high response rates in one cohort study [90]. The role of gastric electrical stimulation in this phenotype is currently being investigated, under the hypothesis that more severe ICC depletion is correlated with lower response rates [91, 92].

#### 4.2.2 High Frequency Phenotype

##### 4.2.2.1 Criteria

Stable high frequency activity as indicated by the Principal Gastric Frequency (refer to Table 1; e.g., Figure 4B).

##### 4.2.2.2 Pathophysiological Considerations

The stomach normally operates within a tight frequency range, with an upper bound of 3.35 cpm for the normative Principal Gastric Frequency range [38, 42]. The Working Group observed that small frequency elevations of ~0.25 cpm may occur incidentally, including during the luteal phase of the menstrual cycle or with progesterone‐based contraceptives, such that borderline or transient frequency abnormalities may be inconsequential and should be interpreted cautiously [93, 94]. In addition, low frequency (< 2.65 cpm) was considered an emerging phenotype only, with clinical relevance currently uncertain, and is discussed subsequently in the manuscript.

Autonomic innervation is known to modulate gastric frequency (although specific linkages require further resolution) [38, 95], and the High Frequency Phenotype has most commonly been observed in the context of presumed vagal neuropathy or injury [35], although it can also be idiopathic. In patients with long‐term type 1 diabetes, Xu et al. found that 28% of participants exhibited abnormally high gastric frequencies, typically being those with more severe chronic GI symptoms and having established peripheral neuropathy and higher HbA1c [35]. In these patients, gastric frequency deviation (absolute difference between PGF and 3.0 cpm) was positively correlated with bloating, upper gut pain, nausea, vomiting, and excessive fullness, as well as total symptoms and reduced quality of life. In a separate study, Xu et al. further investigated gastric myoelectrical abnormalities in a small cohort of patients experiencing persistent symptoms following fundoplication [96]. Elevated frequencies were observed in those with presumed vagal nerve injury (observed at the time of surgery), again with positive correlations between frequency deviation and symptom severity.

This emerging evidence therefore suggests that the High Frequency Phenotype could serve as an indicator of severe vagal nerve dysfunction, although the mechanism, sensitivity, and specificity of this association remain to be determined. This insight is consistent with evidence from low‐resolution EGG studies, which similarly demonstrated persistent elevations in gastric frequency in subsets of patients following vagotomy and has additional support from studies in animal models [97, 98, 99]. This phenotype is observed in ~10% of gastroduodenal patients (Table S3), while noting the significant prevalence modifiers of diabetes and post‐surgical presentations [34].

##### 4.2.2.3 Therapeutic Research Directions

Based on limited data, clinical experience, and associations with diabetic neuropathy, members of the Working Group consider the High Frequency Phenotype as a possible emerging indicator of a treatment‐refractory disease. This position has early support from initial observations in patients referred for GPOEM, which reported a 0% response rate among high‐frequency patients and significant overall differences in gastric frequency between responder and non‐responder groups [100]. A multi‐center prospective study is therefore in progress to validate and extend these preliminary findings (NCT06381349).

Given the emerging associations with neuropathy or vagal nerve injury in this phenotype, a plausible therapeutic approach, particularly in patients at high risk, such as long‐term diabetics, may therefore be directed at the control of neuropathic symptoms [34, 35]. Multidisciplinary care incorporating dietitians, pain services, as well as diabetologists to optimize glycemia should be prioritized, and as a research opportunity, pharmacological considerations could encompass peripheral neuromodulator‐based pharmacotherapy or gabapentinoids [101]. Given the putative association with neuropathy, escalation in therapy for treatment‐refractory patients might also include non‐invasive vagal nerve stimulation [102, 103], or gastric electrical stimulation, particularly when nausea and vomiting are predominant [104, 105]; however, a paucity of current data necessitates further research before clinical recommendations can be provided. Further research is also needed to determine whether specific subgroups could benefit from revisional gastric drainage procedures, and whether acupuncture techniques may have a valid therapeutic role [106].

Importantly, the Working Group notes that this group appears distinct from patients with systemic autonomic dysfunction. In a series of 80 patients treated at a tertiary US center, generalized dysautonomia in the domains of cardiovagal function, axonal sudomotor function, and postural orthostatic tachycardia was associated with delayed gastric emptying, but was not associated with spectral abnormalities such as elevated gastric frequencies [107]. If autonomic contributors are suspected, pancreatic polypeptide sham feeding test of vagal function or direct autonomic function testing may be pursued.

#### 4.2.3 Low Meal Response Phenotype

##### 4.2.3.1 Criteria

Low and/or delayed gastric activity in response to a meal, as indicated by Meal Response Ratio (MRR) and BMI‐Adjusted Amplitude, and meal induced symptoms (refer to Table 1; e.g., Figure 4C).

##### 4.2.3.2 Pathophysiological Considerations

The Low Meal Response Phenotype is characterized by relatively weak or delayed neuromuscular reaction immediately after the meal, in the presence of a normal gastric rhythm, often seen in association with post‐prandial distress. A delayed meal response may follow in the third hour, often correlating to the abatement of symptoms (e.g., Figure 4C) [34]. This phenotype may be present despite the spectral metrics being within reference intervals, standing in contrast with spectral abnormality‐based phenotypes (e.g., concomitant Dysrhythmic Phenotype), which are associated with more severe post‐prandial distress [108]. In a study of 151 patients, this phenotype was seen in 11.2% of patients with normal gastric emptying, and 25% of patients with delayed emptying (also refer Table S3) [12]. Note that high‐amplitude responses were considered an emerging phenotype with uncertain clinical relevance at this time and are discussed subsequently in the manuscript.

To objectively quantify this phenotype, Varghese et al. recently introduced the MRR, which as noted above, is currently calculated as the average amplitude in the first 2 h postprandially divided by the following 2 h. In normal tests, gastric amplitude in the first 2 h generally exceeds that of hours 3–4 (MRR > 1.0). While the Technical Group considered several approaches to quantify this phenotype, including reduced overall amplitudes (e.g., < 22 μV), an MRR < 1.0 was considered most suitable at the current time (refer to discussion) [12]. This decision was based on emerging data linking MRR < 1.0 with delayed gastric emptying [12]. However, qualitative observations of spectrograms remain informative; specifically, relatively low amplitudes in the first two post‐prandial hours (i.e., < 30 μV) may convey therapeutic significance even if the strict ratio criteria are borderline, based on limited data [84].

In a first cohort study implementing this metric, 42.9% of patients with MRR < 1 had delayed gastric emptying, compared to 16.7% of those with a normal MRR [12], possibly caused by proximal fundic retention [108]. An additional possible contributor to a Low Meal Response Phenotype is comorbid constipation, which can suppress post‐prandial gastric activity and delay meal responses without altering rhythm stability via cologastric reflexes [109].

##### 4.2.3.3 Therapeutic Research Directions

This phenotype is commonly observed across adult patients with gastroparesis, FD, and nausea and vomiting disorders [12]. Consistent with these observations, Humphrey et al. also identified this phenotype in approximately one‐third of adolescent patients with FD or gastroparesis, again with associated symptom correlations to the first two post‐prandial hours [44]. Overall, the Low Meal Response Phenotype is therefore considered to offer a diagnostic aid to identify post‐prandial hypomotility, although specific motility correlates are still being evaluated [77].

Emerging real‐world evidence recently demonstrated that the Low Meal Response Phenotype experienced the best symptomatic response to prokinetics [84]. Of note, these patients had BMI‐adjusted amplitudes at the lower end of the normative ranges in the first two post‐prandial hours [42], and lower than those of prokinetic non‐responders. This finding requires further verification and is being actively investigated in an ongoing multicentre cohort study (NCT06854120). Given evidence of weak myoelectrical activity immediately after the meal and association with delayed emptying [12], it is hypothesized that this phenotype may be responsive to low volume, easily digestible, low residue diets to promote transit [80, 81]. However, it is acknowledged that research linking dietary options to clinical outcomes is currently lacking across all phenotypes, and previous inconsistent findings may stem from the heterogeneity of more coarsely defined patient populations. Early evidence also suggests the timing of the meal response may be modulated through simple, non‐invasive vagally‐directed therapies such as resonance breathing (slow diaphragmatic breathing to increase heart rate variability and activate the parasympathetic nervous system) or non‐invasive vagal nerve stimulation [110, 111], which has also been shown to alleviate myoelectrical abnormalities induced by a high gastric volume load [103].

As noted above, clinical members of the Working Group have also observed a high prevalence of comorbid constipation in this group, which may compound delayed meal responses [109], although no published studies have reported this association to date [112, 113]. Management of constipation may therefore present another actionable therapeutic option when the Low Meal Response Phenotype is detected. Further study of this correlation for potential causality needs to be explored.

### 4.3 Auckland Classification: Sensory‐Predominant Phenotypes

#### 4.3.1 Sensorimotor Phenotype

##### 4.3.1.1 Criteria

Symptoms tightly correlated to the gastric amplitude as supported by a high correlation score (> 0.5) (refer to Table 1; e.g., Figure 4D).

##### 4.3.1.2 Pathophysiological Consideration

The Sensorimotor Phenotype indicates cases with stable rhythmic activity, with the critical distinction that symptom curves correlate with gastric myoelectrical activity (i.e., increasing and decreasing in unison with a correlation score > 0.5). Together, these associations indicate that gastric motility has an integral relationship with symptom genesis in the Sensorimotor Phenotype, which is therefore a plausible indicator of gastric hypersensitivity (Figure 4D) [34]. This phenotype is seen in ~25% of patients (Table S3).

GI hypersensitivity can be associated with past enteric infections, immune activation, dysbiosis, and intestinal permeability to chemicals (e.g., acid) and luminal antigens [114], and in females, estrogen‐mediated effects on enterochromaffin cells may be contributory [115]. It is proposed that mechanical stimuli, including distension and contractions trigger hypersensitized viscerosensory pathways [115, 116, 117]. Symptoms typically are meal‐induced, correlate with gastric activity, and subside as the stomach empties and motility wanes; although mixed symptom profiles are also observed in some patients, indicating that overlapping phenotypes should be considered. Interestingly, a recent cohort study of 258 patients showed that this phenotype correlates strongly with higher anxiety scores (although not stress or depression), indicating a gut‐brain link [88]. This finding is consistent with other studies linking chronic GI hypersensitivity disorders to anxiety and hypervigilance [118]. On recent database analysis, ~9% of patients with chronic gastroduodenal symptoms exhibited this phenotype, but higher rates have been observed in different populations [34, 88].

##### 4.3.1.3 Therapeutic Research Directions

Given the putative link to visceral sensitization, promising treatment directions in this phenotype may also be focused on avoidance of trigger foods and neuromodulation medications. Estrogen‐containing contraceptives that are plausibly linked to increased visceral sensitivity could be substituted for alternative contraceptive methods in women [94, 119]; however, further research is required to confirm the benefit of this approach. Similarly, NSAIDs are linked to increased gastroduodenal permeability [120, 121], allowing luminal antigens to trigger inflammation and sensitize visceral afferents [122, 123]. A therapeutic approach to improve barrier dysfunction and restore mucosal integrity, such as avoiding NSAIDs or trialing PPIs, may reduce further sensitisation [124]. Again, the Working Group acknowledges further research is needed in this area. Buspirone could also be considered when impaired accommodation is suspected [125], while pain‐targeted neuromodulators, including tricyclic antidepressants (TCAs), mirtazapine, or serotonin‐norepinephrine reuptake inhibitors (SNRIs), might be beneficial when pain symptoms show a sensorimotor correlation [101, 126]. Importantly, neuromodulators should be individualized to dominant symptoms, including favoring selective serotonin reuptake inhibitors (SSRIs) when comorbid anxiety is present, with expert involvement if possible [101]. Non‐pharmacological interventions, including psychological support such as cognitive behavioral therapy and gut‐directed hypnotherapy, are also worthwhile research directions that could offer additive benefit [127, 128].

Two recent studies raise the hypothesis that therapies directed at gastric emptying may be relevant in the Sensorimotor Phenotype. In one study, Kumar et al. showed a strong relationship between gastric emptying rate and sensorimotor symptoms (not observed for other phenotypes), while Ayubi et al. demonstrated an 80% GPOEM response rate [100, 129]. These studies indicate that further research into the potential role of prokinetics and pylorus‐directed therapies in these patients is warranted.

#### 4.3.2 Continuous Phenotype

##### 4.3.2.1 Criteria

Stable symptom profiles independent of meal or gastric amplitude (refer to Table 1; e.g., Figure 4E).

##### 4.3.2.2 Pathophysiological Considerations

This phenotype is characterized by normal gastric spectral activity, but with a high continuous symptom burden. Symptoms must be elevated, remain stable throughout the test duration (i.e., within a 3‐point range out of 10), and not correlate with the meal or gastric amplitude, implying that gastric myoelectrical activity, as measured by BSGM, does not contribute here to changes in symptoms [34]. The continuous pattern is common, observed in > 20% of patients, regardless of gastroparesis, FD, or chronic nausea and vomiting diagnoses (Table S3) [34, 49].

The Continuous Phenotype has demonstrated consistent group‐level associations with psychological comorbidities, including higher rates of depression, stress, and anxiety, indicating plausible linkage to a centrally‐mediated sensitization etiology in some patients [20, 49, 88]. Centrally‐mediated linkages within the Continuous Phenotype are further supported by emerging data showing worse sleep and more severe illness perceptions (including higher perceived consequences, identified experience, concern, and emotional response—unpublished work).

##### 4.3.2.3 Therapeutic Research Directions

Given that symptoms in the Continuous Phenotype do not correlate with meal ingestion, motility indices, or gastric emptying [129], the Working Group noted that exclusion of organic disease remains an important consideration in patients with continuous symptoms, regardless of psychological comorbidities, and particularly when presentations are atypical (e.g., symptoms of rapid onset, older ages, or accompanied by recent weight loss). In the absence of organic pathologies, the Continuous Phenotype is otherwise considered a plausible indicator of centrally‐mediated symptoms/central sensitization in selected patients, based on the key features discussed above; that is, normal motility [130], meal‐unrelated symptoms [34, 131], high psychological comorbidity [34, 88]; and cognitive associations (unpublished data). This is particularly true when an individual patient's AGBW screening scores are supportive. However, members of the Working Group also noted the need for further research into other potential contributors to symptoms in individuals with the Continuous Phenotype, including immune activation with high circulating levels of inflammatory mediators, and bacterial overgrowth states, to avoid unduly grouping these patients into a common mechanism at this time [132].

Specific therapeutic options in patients exhibiting the Continuous Phenotype are under evaluation, with limited data revealed on literature review. However, in view of the above considerations, lifestyle modification including sleep optimization, regular exercise, avoidance of triggers, and stress management are currently deemed important [133, 134, 135, 136]. Specialist psychological or psychiatric evaluation and targeted interventions also warrant consideration when central sensitization is clinically considered and AGBW scores are high, based on which psychological axis is most affected [27, 137]. Cessation or modulation of hormonal contraception triggers could also be trialed, owing to the emerging marked relationship between estrogen, GI sensitization, and more severe chronic GI symptoms [93, 94, 115, 119], as well as known associations between progesterone and nausea and vomiting [93, 138]. In selected cases, and with expert guidance, this group may additionally benefit from SSRIs [101] and advanced brain‐gut directed therapies, for example, cognitive behavioral therapy or gut‐directed hypnotherapy [127, 128].

Together with holistic patient care strategies, targeted neuromodulation options for specific symptoms are also considered relevant, based on existing guidelines for chronic gastroduodenal disorders. Generally, in patients with gastroduodenal symptom disorders, SSRIs have been recommended when depression or anxiety symptoms are predominant, TCAs or SNRIs when pain is predominant, and tetracyclic antidepressants when early satiation, nausea, vomiting, and weight loss are present [85, 101, 139]. In other guidance, TCAs have been recommended if global foregut symptoms are treatment‐refractory or there is evidence of comorbid irritable bowel syndrome [140]. Notably, the use of TCAs and other anticholinergic medications should be balanced against their side effects, including sedation and constipation [101].

### 4.4 Auckland Classification: Other Phenotypes

#### 4.4.1 Delayed Onset Symptoms Phenotype

##### 4.4.1.1 Criteria

Symptoms predominantly emerge after the majority of postprandial gastric activity is complete (refer to Table 1; e.g., Figure 4F).

##### 4.4.1.2 Pathophysiological Consideration

The Delayed Onset Symptoms Phenotype describes symptoms that occur after the majority of the gastric meal response cycle (timing indicated by MRR ≥ 1), suggesting a small bowel contribution to symptom genesis [28, 34, 57]. This concept is supported by gastric emptying studies showing that certain symptom profiles are sustained after the majority of the meal has exited the stomach [57]. This pattern could therefore plausibly implicate small bowel pathology such as duodenal eosinophilia, hypersensitivity [141, 142], aberrant neuronal or hormonal feedback loops [143], bacterial overgrowth [144], dumping syndromes (including following foregut surgery) [145], and/or small bowel dysmotility [146]; however, further research is required to directly confirm these associations. This phenotype may include motor and/or sensory contributors.

##### 4.4.1.3 Therapeutic Research Directions

This pattern was less common (Table S3). In one study of 210 patients, it was seen in 4.3% where a previous hierarchical phenotyping framework was applied [34], but when seen, it typically associates with pain, bloating, and burning symptomatology [34, 57]. Due to the plausible associations with small bowel activity as noted above, this phenotype warrants additional diagnostic work up focusing on small intestinal pathologies.

### 4.5 Emerging Phenotypes

Additional phenotypes have been proposed and assessed in the literature [34, 58]. While the Working Group did not find sufficient evidence to include these phenotypes in the current core v1.0 set, they represent key areas for future research and evaluation, and likely hold unresolved clinical potential such that they may be included in future versions. Their presence is therefore indicated in the Auckland Classification's automated algorithm to provide clinical insights and standardize future research (refer Appendix D and Table 1 for proposed thresholds). The two most promising emerging phenotypes are:

- “Low Frequency” Phenotype: tests with frequencies below the normative range are observed infrequently [34, 58], except that very low frequencies (2.0–2.3 cpm) are typical after sleeve gastrectomy or other gastric surgeries involving resection of the native pacemaker region, including some esophagogastrectomy procedures [145, 147]. The dominant pacemaker appears to typically remodel, often with reduced stability and frequency [148, 149]. However, a lack of identified symptom or clinical correlations in the evaluated literature concerning low frequencies means that clinical utility was currently deemed speculative
- “High Amplitude” Phenotype: High amplitude activity is observed more commonly and could reflect relative gastric hyperactivity, but motility correlates are currently unknown. Members of the Working Group noted that this finding may be observed if BSGM is performed in patients with gastrostomy tubes, where the stomach wall is pulled up very close to the electrode array, potentially giving an artifactually high voltage reading. However, high amplitude may also be seen in patients without tubes, with several Working Group members pointing to plausible links to distal resistance to flow (e.g., pyloric dysfunction), based on previous low‐resolution EGG studies [150, 151]. However, it is worth noting that the lack of BMI‐adjustment of low‐resolution EGG amplitudes may confound this association, as may medication‐related effects on amplitude (as seen in esophageal manometry, see [152]). Further research is now required to resolve these potential associations and any implications for pyloric therapies.

It is important to note that BSGM does not evaluate several important elements critical to gastric function and symptoms, including fundic accommodation, pyloric function, and duodenal immune activation. Patient reported symptom type and severity in relation to the meal stimulus given during BSGM testing highlight whether symptoms are meal‐responsive or meal‐unrelated and will provide additional data that may point to alternative causes [26, 34, 131]. In conjunction with mental wellbeing screening tools, these data might enable treatment strategies to be matched to current standard of care approaches, such as the Rome criteria for gastroduodenal disorders of gut‐brain interaction [56].

## 5 Discussion

This consensus paper presents the Auckland Classification v1.0, establishing an inaugural standardized framework for the interpretation of BSGM for patients with chronic gastroduodenal symptoms in clinical practice. Based on evidence synthesized from 50 published studies and a dataset of over 4500 clinical tests, the Working Group has defined six principal phenotypes together with an algorithmic workflow for application of the system in practice. These definitions aim to standardize reporting and research methodologies, facilitating the ongoing transition of BSGM from technical validation to clinical application. As with the evolution of the Chicago Classification [30], the first version of this diagnostic framework is intended as an iterative baseline that will be subject to refinement as the evidence base matures.

Current diagnostic labels, such as functional dyspepsia and gastroparesis defined by the Rome Criteria and the results of gastric emptying studies, frequently overlap, lack specificity in terms of disease mechanisms, and do not satisfactorily predict responses to pharmacological or other interventions with certainty [4, 6, 34]. The aim of the new Auckland Classification is to phenotype patients with chronic gastroduodenal symptoms into discrete and biologically plausible mechanistic subgroups based on BSGM technology. By categorizing patients into Motor Predominant (Dysrhythmic, High Frequency, Low Meal Response), Sensory Predominant (Sensorimotor, Continuous), and Delayed Onset Symptoms Phenotypes, this classification aims to provide additional objective and actionable biomarkers for mechanisms that were previously described but difficult to identify using existing tests in clinical practice [9]. For example, the Dysrhythmic Phenotype presents a plausible non‐invasive surrogate for the ICC depletion and associated neuromuscular dysfunction that is known to underpin a spectrum of GI dysfunction [4, 60, 62], while the Sensorimotor Phenotype offers a plausible indicator of gastric hypersensitivity, and the Continuous Phenotype supports central sensitization in selected patients with risk factors and indicators [20, 34]. Using BSGM to delineate subgroups that respond to distinct therapeutic approaches would be a major advance in the management of patients with these challenging conditions. Importantly, this overarching hypothesis can be tested. Validation in prospective studies is now essential, with early data suggesting that phenotype‐guided management, such as prokinetics for the Low Meal Response Phenotype, GPOEM for the Dysrhythmic Phenotype, or neuromodulators for the Sensorimotor Phenotype, may improve therapeutic precision and outcomes compared to empiric trial‐and‐error [71, 84, 90, 153].

The integration of BSGM into clinical workflows therefore has potential to address key limitations of current testing modalities. While 4‐h gastric emptying scintigraphy (GES) remains the standard for assessing transit, it does not elucidate the mechanism of delay and therefore does not provide sufficiently specific therapeutic targets, nor does it identify pathology in patients with normal emptying [4, 9]. Moreover, with the exception of the Nottingham Test Meal protocol [154, 155], existing methods for assessing gastric emptying do not assess gastric sensation by recording patient symptoms during gastric filling or emptying. The Working Group views BSGM as complementary to GES and other diagnostics that assess transit time (i.e., wireless motility capsule, breath testing), with their combined use allowing for the differentiation of gastric myoelectrical dysfunction from transit delays, resulting in an overall substantially higher combined diagnostic yield for any gastric motility abnormality [12, 44, 49]. Importantly, the Auckland Classification's phenotypes go beyond some of the limitations of gastric emptying testing alone, by achieving superior and more specific symptom and psychological correlations across broad disease categories as reviewed here. The two tests may also be performed synchronously in practice, so long as meals can be successfully harmonized [12, 49]. This enhanced mechanistic clarity supported the presentation of a provisional, research‐guiding, therapeutic algorithm presented in this review. This algorithm uses current society guidelines to identify the range of available therapeutic options, rather than seeking to reconcile different recommendations between societies (e.g., regarding G‐POEM) [3, 8, 14, 17, 133, 156, 157, 158, 159, 160, 161].

As the first consensus classification for BSGM, this framework has several limitations and is expected to evolve. First, while the phenotypes are supported by translational and clinical cohort studies, direct validation against histopathology (e.g., ICC counts), vagal nerve testing, or motility correlates (e.g., ADM) remain limited and would benefit from expansion [63, 77, 96]. Second, the algorithmic framework for automated implementation of the scheme (Appendix D) currently includes a mix of robust quantitative cut‐offs guided by reference intervals, emerging linkages estimated from current experience and clinical research studies, and those included based on Working Group consensus. We invite the broader GI community to challenge and test this framework to facilitate its continuous improvement. Third, the current classification is derived primarily from adult data; while adolescent studies show consistent patterns, a dedicated pediatric classification is currently in development to account for developmental physiology [31]. Fourth, we have primarily considered the proposed phenotypes in isolation; while the algorithmic framework supports the reporting of mixed phenotypes, the clinical significance of co‐occurring phenotypes is not yet well‐understood. Lastly, emerging phenotypes and important areas for future investigation have been identified, including novel biomarkers such as spatial wave propagation metrics that could be integrated in future as technical capabilities advance [22, 162].

The most challenging phenotype for the technical group to objectively quantify in the Auckland Classification v1.0 was the Low Meal Response Phenotype. The Fed:Fasted Amplitude Ratio (ff‐AR) was not found to be sufficiently discerning, as high fasting baselines are common in both control and patient data [42], and no associations were identified with either symptoms or clinical outcomes on literature review. This finding is also consistent with low‐resolution EGG studies, which generally failed to find group‐level differences in “dominant power” changes between fasting and fed states [163, 164, 165]. The ff‐AR metric may therefore be deprioritised in future [40, 42]. Instead, the MRR was adopted to capture meal response timing. While MRR < 1 correlates with delayed emptying, symptoms, and possibly prokinetic response [12, 44, 84, 108], it lacks specificity in isolation as healthy controls can also exhibit late peaks, despite a normative mean > 1 (1.18 ± 0.42; unpublished data). Furthermore, strict amplitude cut‐offs (< 22 μV) lacked sensitivity, with prokinetic responders often showing higher values up to 30 μV [84]. Therefore, clinical assessment of this phenotype is currently more complex, requiring simultaneous evaluation of meal response profile (as measured by MRR), strength (as measured by BMI‐adjusted amplitude), and symptom severity in response to meals. The inclusion of symptoms was considered necessary to separate this clinical phenotype from the physiological findings that may also occur in healthy controls, and is supported by precedents in the Chicago Classification v4.0 [30]. Further iteration is anticipated as ongoing prokinetic responder studies mature across our consortium (including NCT06854120).

Several additional areas of further investigation were also elucidated by the Working Group during iterative feedback stages. First, the Delayed Onset Symptoms phenotype fell below the consensus threshold of 80%, identifying this as a target area for further validation studies. As this is a relatively low prevalence phenotype, larger series are anticipated to clarify its clinical relevance. Second, several Working Group members identified the occurrence of meal responsive symptoms that do not meet the threshold for a sensorimotor correlation. Further, it is acknowledged that the present BSGM literature is still emerging, and further independent reproduction of early reports is desirable. Further work is required to evaluate patients who do not fall within the current classification, which may lead to threshold adjustments in future versions.

We further emphasize that, as a foundational scheme, the Auckland Classification v1.0 should be viewed as a platform for future development (refer Appendix F, Table S4 for list of ongoing studies). While further elucidation of mechanistic associations, the “emerging” phenotypes, and the expansion of normative data across broader demographic groups are important focus areas, the highest priority work for the next iteration of the Auckland Classification is the ongoing validation of the proposed therapeutic algorithm through prospective trials. We also anticipate that future versions will incorporate increasingly rigorous methodologies, including patient involvement, as the framework matures. The current resource requirements for conducting a standardized BSGM test protocol are also presented in Appendix G, which may change as future methodologies evolve.

In conclusion, the Auckland Classification v1.0 formalizes a standardized clinical nomenclature and system for Body Surface Gastric Mapping. By moving beyond symptom‐based labels toward mechanism‐based phenotyping, this consensus framework provides a structured approach for clinical decision‐making and a foundation for the next generation of research in gastroduodenal disorders.

## Author Contributions

C.V., G.O., A.A.G., G.S., N.D., and D.F. contributed literature review, drafting, and figure preparations. All authors contributed critical review, edits, methodological review, and appraisal.

## Funding

This work was supported by the New Zealand Health Research Council.

## Disclosure

International Body Surface Gastric Mapping Working Group.

Technical Group: Chris Varghese, Nicky Dachs, Gabriel Schamberg, Armen A. Gharibans, Daphne Foong, Mikaela Law, Stefan Calder, Charlotte Daker, Christopher N. Andrews, Greg O'Grady.

Consensus Group: David Levinthal, Baharak Moshiree, Braden Kuo, Catherine Ngo, Sahib S. Khalsa, Charles Verdonk, William L. Hasler, Xiao Jing Wang, Nicholas R. Oblizajek, Thomas Abell, Henry P. Parkman, Linda Nguyen, Ali Rezaie, Irene Sonu, David Kunkel, Jon Erickson, Vincent Ho, Anthony Robert Hobson, Daniel Keszthelyi, Leila Neshatian, Jan Tack, Christopher Brian Cederwall, Bianca Chang, I‐Hsuan Huang, Mohammad Bashashati, Joseph Sujka, Rehan Haidry, Bu'Hussain Hayee, Homira Ayubi, Mohammad Al‐Haddad, Sven Eriksson, Shahin Ayazi, Sanjay Pandanaboyana, Mark Fox, John Clarke, Gerald Holtman, Natasha Koloski, Robert Bulat, Amol Sharma, Joy J. Liu, Douglas Weinstein, Uday Chand Ghoshal, Mahesh Goenka, Asma Fikree, Natalia Zarate‐Lopez, Prateek Mathur, Abigail Stocker, Peng Du, Leo K. Cheng, Timothy R. Angeli‐Gordon, Sam Simmonds, William Xu, Jonathan Sivakumar, John A. Windsor, Jarongkorn Sirimongkolkasem, Nicha Wongjarupong, Alexander Podboy.

## Conflicts of Interest

A.A.G., S.C., G.S., N.D., and G.O. hold grants and intellectual property in GI electrophysiology. Several authors are members of University of Auckland Spin‐out companies: The Insides Company (GO), and Alimetry Ltd. (A.A.G., S.C., G.S., N.D., G.O., C.V., D.F., M.L., S.S., J.W., P.D., C.D., and C.N.A.). The remaining authors have no relevant conflicts.

## Supporting information


**Table S1:** List of studies evaluated by the technical group to inform the Auckland Classification v1.0.
**Table S2:** Proposed algorithmic implementation of Auckland Classification version 1.0. A BSGM test is evaluated against every criteria listed in the table. When one or more criteria is met, the automated classification is determined as the highest priority criteria or criterion.
**Table S3:** Prevalence of Auckland Classification v1.0 phenotypes by gastroduodenal disorder diagnosis.
**Table S4:** List of ongoing body surface gastric mapping studies (excludes completed and published studies).

## Data Availability

The data that supports the findings of this study are available in the Supporting Information of this article.
