# Supplementary material for: Expert Clinical Consensus on Body Surface Gastric Mapping Phenotypes for Gastroduodenal Disorders: ‘Auckland Classification’ v1.0
Source: Neurogastroenterol Motil. 2026 Jul 1;38(7):e70377. doi: 10.1111/nmo.70377 (PMC13320621; doi:10.1111/nmo.70377)
Supplement: Supplementary file 1 — Table S1: List of studies evaluated by the technical group to inform the Auckland Classification v1.0. Table S2: Proposed algorithmic implementation of Auckland Classification version 1.0. A BSGM test is evaluated against every criteria listed in the table. When one or more criteria is met, the automated classification is determined as the highest priority criteria or criterion. Table S3: Prevalence of Auckland Classification v1.0 phenotypes by gastroduodenal disorder diagnosis. Table S4: List of ongoing body surface gastric mapping studies (excludes completed and published studies). [file NMO-38-e70377-s001.docx]

**Supplementary Material** for ‘Expert clinical consensus on body surface gastric mapping phenotypes for gastroduodenal disorders: ‘Auckland Classification’ v1.0’

**Supplementary Appendix A**

Test Quality and Protocol Considerations

The proposed classification scheme and the accompanying automated classification algorithm require consideration of the BSGM test quality and protocol when incorporating the resulting classifications into clinical practice, as multiple aspects may affect the likelihood and/or reliability of the presentation of a particular phenotype.^28^ As with other physiological tests, the incorporation of quality and protocol considerations is not currently included in the proposed automated algorithm, and must be performed manually by the clinical users. Possible quality and protocol considerations, and their effects on interpretation, are reviewed below.

Test quality-related measures (e.g., patient movement, electrode impedance, timing of artifact detection/correction, and patient BMI) and protocol information (e.g., meal type, percentage of meal consumed, test duration) are summarized in the Gastric Alimetry report. Where these measures fall outside of recommended or validated intervals, a caution is automatically raised, advising interpretation with care.^22,28,44,54^ If no cautions are raised, this indicates the test is suitable for classification without specific consideration of these factors.

- **Artifacts Detected:** The Gastric Alimetry system raises a caution when artifact detection >50%, indicating that the signal processing pipeline has substantially altered the majority of the test signals. In many instances, the test remains interpretable, as modern processing techniques have sufficiently improved signal quality.^44^ If artifacts are >50%, but the GA-RI is ≥0.25, the test is likely reliable as stable gastric activity was detected despite the artifacts. If artifacts are >50% and the GA-RI is <0.25, greater caution is necessary. This is particularly true if the periods of high artifact align with the apparent dysrhythmic activity, or occur in the presence of poor impedance or high patient movement. In some instances, artifacts will be identified as uncorrectable and the data will be removed. In such cases, it is important to consider the effects of missing data on the applicability of reference intervals.
- **Electrode Impedance:** The Gastric Alimetry system raises a caution when over 32 channels show poor impedance. This increases the likelihood of artifacts. The test may still be reliable if a sufficient number of remaining channels have a good connection and are largely free of artifacts. Caution is primarily required when interpreting a Low Meal Response phenotype. Poor connection in part of the array may force the algorithm to rely on suboptimally placed electrodes, potentially causing a reduction in signal amplitude that is not reflective of gastric dysfunction.
- **Patient BMI:** The Gastric Alimetry system raises a caution when BMI > 35 kg/m^2^. High BMI increases adipose tissue separating the electrodes from the stomach, which attenuates gastric potentials. This can decrease measured amplitudes and rhythm stability.^28^ However, both the BMI-adjusted amplitude and GA-RI metrics include adjustments to account for this effect.^45^ Consequently, patients with high BMI are *less likely* to have an out-of-reference interval BMI-adjusted amplitude or GA-RI. In some high BMI cases, a PGF may not be detectable even if the GA-RI is ≥0.25. This indicates no apparent rhythmic activity, but this finding is not considered abnormal for a patient with high BMI. Therefore, high BMI limits the sensitivity for detecting the Dysrhythmic and Low Meal Response phenotypes. Tests from patients with high BMI (>35 kg/m^2^) should be visually reviewed. Before assigning a phenotype predicated on stable activity (e.g., High Frequency, Sensorimotor, Continuous), the spectrogram should be inspected to confirm that rhythmic activity is clearly visible.

Protocol considerations relate to factors that may affect the clinical significance of the test outcomes independent of technical signal quality. While many tests with non-standard protocols may present clear clinical findings, any protocol deviation reduces the applicability of the normative reference intervals, as these were determined from a cohort with the standard test protocol.^47^

- **Test Duration:** The Gastric Alimetry system raises a caution when the total test duration is less than 4 hours. This creates a risk that a delayed onset meal response or symptom response may not be captured. If the test ends early and no clear gastric or symptom response has occurred, it must be interpreted with caution. Short tests with clear meal responses and symptom profiles are likely interpretable. It should be noted that a reduced duration may slightly elevate the *overall* amplitude if a typical (tapering) response is cut short, or conversely, lower it if a delayed response is missed.
- **Meal Type and Completion:** The Gastric Alimetry system raises a caution when the patient eats a non-standard meal or consumes < 50% of the meal.These factors can affect the test metrics in multiple ways; for instance, a higher caloric intake may increase the strength and/or duration of the postprandial meal response.^54^ While these factors provide clinically relevant information, their effects on the results must be considered when applying standard classifications. Diabetic, vegan, and gluten-free meals are all considered standard meals for the purposes of the protocol.

**Supplementary Appendix B**

Spectral Metrics

- *Principal Gastric Frequency (PGF) [reference interval 2.65 - 3.35 cpm]*: Principal Gastric Frequency is observed as a dominant horizontal band in the spectrogram, and is reported in cycles per minute (cpm). Normal frequency lies in a tight range (2.65 - 3.35 cpm), with some upwards drift of 0.1-0.2 cpm in the luteal phase of the menstrual cycle or with progesterone-based contraceptives.^98,99^ Reduced frequencies are mainly seen after surgical procedures resecting or affecting the gastric pacemaker region (e.g., sleeve gastrectomy),^9,10^ and may be inconsequential. Higher frequencies (i.e., >3.5 cpm) are often pathological and may lead to increasingly ineffective motility at higher ranges due to a correlated decline in slow wave velocity.^11,12^
- *Gastric Alimetry Rhythm Index (GA-RI^®^)* *[reference interval ≥0.25]*: Gastric Alimetry introduces a ‘rhythm index’ that defines dysrhythmia.^47^ This is quantified by the percentage of power distribution inside the dominant gastric frequency band compared to outside, with higher values indicating greater stability. Rhythm may be *transiently* abnormal, e.g., after excessive gastric distension, with correlations to transient symptoms such as nausea and post-prandial distress.^13,14^ Various pharmacological agents or physiological stimuli such as motion sickness may also transiently affect rhythm.^12,15,16^ However, dysrhythmia that is *sustained* over the majority of a test duration is considered by the Working Group to be indicative of an underlying gastric neuromuscular disorder.
- *BMI-Adjusted Amplitude [reference interval 22-70 μV]*: Gastric potentials are rapidly attenuated by adipose tissue, hence BMI correction allows data to be usefully compared between individuals.^5,6,17^ Low amplitudes are considered to be associated with hypomotility, myopathy and/or neuromuscular dysfunction.^1,18^
- *Fed:Fasted Amplitude Ratio (ff-AR) [reference interval ≥1.08]*: An increase in the signal power after the meal is indicative of a meal response. Because ~25% of controls display a ‘high fasting baseline’ amplitude, potentially related to migrating motor complex activity,^47^ cephalic phase activity,^19^ or circadian variations,^20^ ff-AR has a low reference interval and is considered a supporting metric that should not be used to define gastric dysfunction alone.^20^
- *Meal Response Ratio:* The timing of the postprandial meal response can also be simply quantified through the Meal Response Ratio (MRR),^21^ calculated as the ratio of the average amplitude in the first 2 hrs postprandially to that of the following 2 hrs. MRR is not calculated when less than 2.5 hrs of post-test data is available. MRR can be empirically considered normal at values > 1, indicating that the dominant gastric motor response occurred within the first two hours after a meal.^21^

Symptom-Amplitude Metrics

- *Symptom-Amplitude Correlation Score [strong correlation if score > 0.5]:* The association between gastric amplitude and symptoms over the test duration is reported by a *correlation score* between 0-1, which aims to offer a proxy indicator of gastric hypersensitivity (**Figure 3B**), or the temporal association between gastric motility and symptoms.^22^

**Supplementary Appendix C**

**Table S1:** List of Studies Evaluated by the Technical Group to Inform the Auckland Classification v1.0

| **Study** | **Cohort** | **Study Design** | **Country** | **Sample** | **Other Diagnostic Modalities Compared** | **Therapy Investigated** |
| --- | --- | --- | --- | --- | --- | --- |
| Varghese, Van Hove et al 2025^23^ | Adults with chronic gastroduodenal symptoms | Prospective cohort study | Australia and New Zealand | 42 | - | Prokinetics |
| Varghese, Huang et al 2025^21^ | Adults with chronic gastroduodenal symptoms | Prospective cohort study | Belgium | 143 | Gastric emptying breath test | - |
| Humphrey et al 2025^24^ | - | Narrative review | - | - | - | - |
| Sadaka et al 2025^25^ | Children aged 10-19 with gastroduodenal symptoms | Prospective cohort study | USA | 15 | Antroduodenal manometry | - |
| Gharibans et al 2025^26^ | Adults with chronic gastroduodenal symptoms | Retrospective analysis | Belgium | 109 | Rome IV | - |
| Ayubi et al 2025^27^ | Adults with gastroparesis undergoing GPOEM | Prospective cohort study | UK | 30 | - | GPOEM |
| Wang et al 2025^28^ | Adult patients that have undergone pancreatoduodenectomy | Prospective cohort study | New Zealand | 19 patients (19 matched controls) | - |  |
| Varghese et al 2025^29^ | - | Narrative review | - | - | - | - |
| Shaaban et al 2025^30^ | Adult healthy volunteers | Prospective cohort study | New Zealand | 20 | Liquid nutrient drink test |  |
| Lim et al 2025^30^ | Adults patients with chronic gastroduodenal disorders | Retrospective analysis | Australia, New Zealand and Canada | 127 | - | Hormonal contraception |
| Law et al 2025^31^ | Adults patients with chronic gastroduodenal disorders | Retrospective analysis | Australia, New Zealand and Canada | 278 | - | - |
| Coutinho et al 2025^32^ | Adult healthy volunteers | Prospective cohort study | New Zealand | 20 | Gastric emptying breath test | GLP-1 agonist |
| Xu et al 2025^33^ | Adult patients with being investigated for gastro-oesophageal reflux | Prospective cohort study | New Zealand | 20 patients (20 matched controls) | 24 hour pH study | - |
| Varghese et al 2025^34^ | Adults patients with chronic gastroduodenal disorders | Retrospective analysis | USA | 80 | Formal autonomic testing | - |
| Law et al 2025^35^ | Children aged 12-17 | Prospective cohort study | New Zealand | 33 | - | - |
| Humphrey et al 2025 (2)^36^ | Adolescent patients aged 12-21 with chronic gastroduodenal disorders | Prospective cohort study | New Zealand and USA | 25 patients (31 matched controls) | - | - |
| Wang et al 2025 (2) | Adult patients that have undergone sleeve gastrectomy | Prospective cohort study | Australia, New Zealand and Canada | 38 patients (38 matched controls) | - | - |
| Varghese et al 2025 (2)^37^ | Adults patients with chronic gastroduodenal disorders | Prospective cohort study | Australia and New Zealand | 67 | Gastric emptying scintigraphy with intragastric meal distribution analysis | - |
| Simmonds et al 2025^38^ | Adults | Retrospective analysis | Australia, New Zealand and Canada | 172 | - | - |
| Varghese et al 2025 (3)^22^ | Adults patients with chronic gastroduodenal disorders | Retrospective analysis | Australia, New Zealand and Canada | 210 | - | - |
| Wang et al 2024^39^ | Adult patients that have undergone gastric bypass | Prospective cohort study | New Zealand | 38 | - | - |
| Law et al 2024^40^ | Adults patients with chronic gastroduodenal disorders | Qualitative study | New Zealand | 16 | - | - |
| Huang et al 2024^40^ | Adult healthy volunteers | Retrospective analysis | Australia, New Zealand, Canda, Belgium, USA | 238 | - | - |
| Law et al 2024 (2)^41^ | Adults patients with chronic gastroduodenal disorders | Prospective cohort study | New Zealand | 12 | - | - |
| Law et al 2024 (3)^42^ | Adults patients with chronic gastroduodenal disorders and healthy volunteers | Prospective cohort study | Australia, New Zealand and Canada | 14 patients (14 controls) | - | - |
| Lim et al 2024^42^ | Adult healthy volunteers | Retrospective analysis | Australia, New Zealand and Canada | 121 | - | - |
| Huang et al 2024 (2)^43^ | Adult healthy volunteers | Prospective cohort study | Belgium | 20 | Gastric emptying breath test and subset (n=11) with barostat | Corticotropin-releasing hormone |
| Humphrey et al 2024^44^ | Children aged 5-18 | Qualitative study | New Zealand | 123 | - | - |
| Xu et al 2024^45^ | Adult patients that have undergone fundoplication | Prospective cohort study | Australia and New Zealand | 16 patients (16 matched controls) | - | - |
| Wang et al 2024^46^ | Adults patients with chronic gastroduodenal disorders | Retrospective analysis | Australia and New Zealand | 75 | Gastric emptying scintigraphy | - |
| Du et al 2024^46^ | Adult healthy volunteers | Prospective cohort study | New Zealand | 18 | Water load satiety test | Transcutaneous auricular vagus nerve stimulation |
| Foong et al 2023^47^ | - | Narrative review | - | - | - | - |
| O'Grady et al 2023^47^ | - | Narrative review | - | - | - | - |
| Schamberg et al 2023^48^ | Adults patients with chronic gastroduodenal disorders and healthy volunteers | Prospective cohort study | Australia, New Zealand, Canda, USA | 178 | Legacy electrogastrography | - |
| Wang et al 2023^152^ | Adult patients that have undergone oesophagectomy | Prospective cohort study | New Zealand | 6 | - | - |
| Varghese, Schamberg et al 2023^17^ | Adult healthy volunteers | Prospective cohort study | Australia, New Zealand, Canda, USA | 110 | - | - |
| Schamberg et al 2023 (2)^47^ | Adult healthy volunteers | Retrospective analysis | New Zealand | 100 | - | - |
| Gharibans et al 2023^48^ | Adult healthy volunteers | Prospective cohort study | New Zealand | 24 | - | - |
| Xu et al 2023^49^ | Adult patients with type 1 diabetes mellitus | Prospective cohort study | New Zealand and Canada | 32 patients (32 matched controls) | - | - |
| O'Grady et al 2022^50^ | - | Narrative review | - | - | - | - |
| Calder et al 2022^50^ | Adults patients with chronic gastroduodenal disorders | Prospective cohort study | New Zealand | 10 | - | - |
| Calder et al 2022 (2)^51^ | Porcine | Animal study | New Zealand | 14 (porcine) | High-resolution serosal mapping | - |
| Gharibans et al 2022^51^ | Adults patients with chronic gastroduodenal disorders | Prospective cohort study | New Zealand and Canada | 43 patients (43 matched controls) | - | - |
| Sebaratnam et al 2022^52^ | Adults patients with chronic gastroduodenal disorders | Retrospective analysis | New Zealand and Canada | 79 | - | - |
| Carson et al 2021^53^ | - | Narrative review | - | - | - | - |
| Somarajan et al 2021^54^ | Children aged 12-17 with chronic gastroduodenal disorders | Prospective cohort study | USA | 10 | - | - |
| Gharibans et al 2019^55^ | Adults patients with chronic gastroduodenal disorders and healthy volunteers | Prospective cohort study | USA | 25 patients (7 controls) | - | - |
| O'Grady et al 2019^56^ | - | Narrative review | - | - | - | - |
| Gharibans et al 2018^56^ | Children aged 7-17 with chronic gastroduodenal disorders | Prospective cohort study | USA | 11 | Antroduodenal manometry | - |
| Gharibans et al 2017^57^ | Adult healthy volunteers | Prospective cohort study | USA | 8 | - | - |

**Supplementary Appendix D**

Automated Auckland Classification Algorithm

In addition to the core phenotype set, the Technical Group further proposed an algorithmic method for automatically determining the prominent phenotype(s) and/or other clinically relevant information from the quantitative outputs of a BSGM test, aligned with the Auckland Classification v1.0. As with the phenotypes, the classification algorithm was developed through consensus agreement and review of clinical BSGM test results and relevant literature. The quantitative framework for automatically determining the phenotype of a BSGM test aims to enable reproducibility whilst retaining nuance in BSGM interpretation.

The algorithm processes a defined set of quantitative outputs from the Gastric Alimetry test report. These inputs include:

- **Standard Spectral Metrics**: The four primary biomarkers detailed above (Gastric Alimetry Rhythm Index (GA-RI), Principal Gastric Frequency (PGF), BMI-Adjusted Amplitude, and the supporting Meal Response Ratio (MRR).
- **Symptom Subgroup Scores**: Quantitative scores (ranging from 0-10) that group symptoms into four clinically recognized categories: ‘Nausea & Vomiting’, ‘Pain’, ‘Postprandial Distress’, and ‘Burning & Reflux’.^26^
- **Symptom Tags**: Descriptive, rule-based tags that identify common temporal symptom patterns. These include ‘Meal Induced’, ‘Meal Alleviated’, ‘Late Onset’, and ‘Continuous’. A separate ‘Sensorimotor’ tag is applied when a symptom shows a high correlation (>0.5) with the BMI-Adjusted Amplitude curve (discussed in full here ^26^).
- **Gut-Brain Wellbeing (AGBW) Scores**: The subgroup scores (depression, stress, and anxiety) from the validated Alimetry Gut-Brain Wellbeing (AGBW) Survey.

The symptom subgroup scores, symptom tags, and AGBW subgroup scores are new features to the Gastric Alimetry test that have recently been cleared by the FDA.^57–59^

The classification algorithm operates as a prioritized hierarchy, evaluating test outputs against the set of criteria defined in **Table S2**. The classification is determined by the highest priority criterion (Priority 1 being highest) that is met. For instance, a severely abnormal rhythm will always result in a 'Dysrhythmic' classification, regardless of whether criteria for other, lower-priority phenotypes are also met. In setting these criteria within an algorithmic framework, categorical judgements were necessarily applied by the Technical Group, based on a combination of the literature review and database experience from >4,500 banked cases. It is accepted that some of these decisions reflect best current estimates to allow arrival at an initial objective framework suitable for iterative refinement as further research emerges.

This hierarchical structure is designed to handle mixed evidence and to encode the strength of a finding. Criteria at higher priorities (e.g., Priority 1) are therefore intended to represent more definitive or clinically significant evidence for a phenotype than those at lower priorities (e.g., Priority 3 or 4). The algorithm can also report combined phenotypes if multiple criteria are met at the same highest-priority level. For example, at Priority 2, a test meeting the criteria for both 'High Frequency' (PGF > 3.4) and 'Sensorimotor' (2+ symptoms with Sensorimotor tags) would be classified as a combination of both if the GA-RI was above 0.22 (see logic below). Finally, this framework naturally extends to commenting on other potentially clinically relevant test outputs (e.g., emerging phenotypes) when none of the core six phenotypes are detected at a higher priority.

The priority structure in **Table S2** is therefore designed to classify the most definitive and clinically significant findings first, before considering sensory-dominant patterns, or less definitive abnormalities.

- **Priority 1**: This level is reserved for the most robust biomarker of significant neuromuscular dysfunction: a severely abnormal rhythm (GA-RI < 0.22). This finding is considered to be of primary clinical importance and thus supersedes all other potential classifications, as the other motor-dominant phenotypes (high frequency and low meal response) are phenotypes that are only relevant in the presence of coordinated rhythmic activity. The use of 0.22 as the cutoff for the higher priority determination of the dysrhythmic phenotype is motivated by the lower end of the 90% confidence interval for the reference interval cutoff of 0.25 for GA-RI (unpublished data).
- **Priority 2**: This level captures borderline abnormal rhythm stability (0.22 ≤ GA-RI < 0.25), frequency activity in the top 2.5 percent of controls (PGF > 3.4), a meal response that is weaker in the immediate postprandial period than the end of the test (MRR < 1; supported by meal-responsive symptoms), or a strong indication of a sensory-dominant symptom profile appearing in multiple symptoms (3+ tags for Continuous, 2+ tags for Sensorimotor or Delayed Onset).
- **Priority 3**: This level is used for weaker indications of the main phenotypes in the presence of a stable gastric activity (GA-RI ≥ 0.25). The criteria at this level for motor-dominant phenotypes are aligned with the normative reference intervals for healthy controls, with high frequency determined as PGF > 3.35 cpm and weak meal response determined as BMI-adjusted amplitude < 22 µV (overall or in either of the first two postprandial hours; supported by meal-responsive symptoms). For the sensory-dominant phenotypes, lower priority evidence is considered the presence of just one or two symptom tags. These weaker phenotype criteria overlap in priority with mental health symptoms that are experienced most or all of the time for a specific subgroup (anxiety, stress, or depression), as measured by the AGBW survey, as well as a low meal response (MRR <1) that is not supported by meal-responsive symptoms. As the AGBW survey only provides a screening measure of mental health, high scores are only included in the automated classification when there is only weak (or no) evidence of alternative motor or sensory phenotype.
- **Priorities 4 & 5**: These lower priorities are used to determine other information in the report that may yield clinically relevant information in the absence of evidence for a dominant phenotype. This information includes the gut-brain wellbeing survey results, out-of-reference interval spectral metrics, symptom tags, and symptom subgroup scores.

**Table S2:** Proposed algorithmic implementation of Auckland Classification version 1.0. A BSGM test is evaluated against every criteria listed in the table. When one or more criteria is met, the automated classification is determined as the highest priority criteria or criterion.

|  | *Motor Predominant Phenotypes* | | | *Sensory Predominant Phenotypes* | | *Small Bowel Phenotypes* | *Relevant clinical information when no phenotype* | | | | | | | |
| --- | --- | --- | --- | --- | --- | --- | --- | --- | --- | --- | --- | --- | --- | --- |
| ***Phenotype* (across)**  ***Priority* (down)** | ***Dysrhythmic*** | ***High Frequency*** | ***Low Meal Response*** | ***Sensorimotor*** | ***Continuous*** | ***Delayed Onset Symptoms*** | ***Low Meal Response Ratio*** | ***Gut-Brain*** | ***High Amplitude*** | ***Low Amplitude*** | ***Low Frequency*** | ***Meal Induced*** | ***Meal Alleviated*** | ***Symptom Subgroup Scores*** |
| ***1*** | GA-RI < 0.22 |  |  |  |  |  |  |  |  |  |  |  |  |  |
| ***2*** | 0.22 ≤ GA-RI < 0.25 | PGF > 3.4 | MRR <1 and 1+ Meal Induced tags | 2+ tags | 3+ tags | 2+ tags and MRR ≥ 1 |  |  |  |  |  |  |  |  |
| ***3*** |  | 3.35 < PGF ≤ 3.4 | Adjusted amplitude < 22 µV overall or in either first or second postprandial hours, and 1+ Meal Induced tags | 1 tag | 1 or 2 tags | 1+ tags | MRR < 1 | One or more subscale >= Most of the time |  |  |  |  |  |  |
| ***4*** |  |  |  |  |  |  |  | One or more subscale >= Some of the time | Adjusted Amplitude > 70 µV | Adjusted Amplitude < 22 µV | PGF < 2.65 | 2+ tags | 2+ tags | Max subscore > 5 |
| ***5*** |  |  |  |  |  |  |  |  |  |  |  | 1 tag | 1 tag | Max subscore > 2 |

**Supplementary Appendix E**

Phenotype Prevalence

The prevalence of each phenotype were reported with stratification by gastroparesis, functional dyspepsia, and chronic nausea and vomiting syndrome diagnoses based on Rome IV and established gastric emptying test cut-offs (see ^58–60^). This cohort encompassed 176 participants from previous studies (median age 40.5 years, 79.0% female).^21,37,46^ Patients could have multiple phenotypes. These cohorts included patients that underwent gastric emptying breath testing (Leuven, Belgium cohort; ethical approval: S65541) and gastric emptying scintigraphy with ~255 kcal low fat egg meal (Auckland, New Zealand cohort and Western Sydney, Australia cohort; ethical approvals AHREC123 and H13541). It is noted that phenotype prevalence is expected to vary based on the referral population.

In this multicenter cohort of 176 participants (median age 40.5 years, 79.0% female), 165 met criteria for a chronic gastroduodenal disorder and had complete BSGM tests for phenotyping (3 excluded for post-prandial duration <2.5 h, 8 excluded for not meeting criteria for a chronic gastroduodenal disorder). Using Rome IV, 75 (44.5%) had functional dyspepsia, 7 (4.2%) had chronic nausea and vomiting syndrome and 67 (40.6%) met criteria for both. The remaining 16 (9.7%) had gastroparesis based on delayed gastric emptying breath testing or scintigraphy. Of these 165, 125 (75.8%) were classified by the Auckland Classification v1.0: 20 (12.1%) met criteria for Dysrhythmic Phenotype, 16 (9.7%) met criteria for High Frequency Phenotype, 13 (7.9%) met criteria for Low Meal Response Phenotype, 43 (26.1%) met criteria for Sensorimotor Phenotype, 45 (27.3%) met criteria for Continuous Phenotype, and 6 (3.6%) met criteria for the Delayed Onset Symptoms phenotype.

**Table S3**: Prevalence of Auckland Classification v1.0 phenotypes by gastroduodenal disorder diagnosis

|  | CNVS  (n=7) | FD  (n=75) | FD+CNVS  (n=67) | Gastroparesis  (n=16) | Overall  (n=165) |
| --- | --- | --- | --- | --- | --- |
| Unclassified | 2 (28.6%) | 21 (28.0%) | 14 (20.9%) | 3 (18.8%) | 40 (24.2%) |
| Dysrhythmic Phenotype | 2 (28.6%) | 11 (14.7%) | 3 (4.5%) | 4 (25.0%) | 20 (12.1%) |
| High Frequency Phenotype | 1 (14.3%) | 9 (12.0%) | 6 (9.0%) | 0 (0%) | 16 (9.7%) |
| Low Meal Response Phenotype | 0 (0%) | 4 (5.3%) | 6 (9.0%) | 3 (18.8%) | 13 (7.9%) |
| Sensorimotor Phenotype | 3 (42.9%) | 20 (26.7%) | 16 (23.9%) | 4 (25.0%) | 43 (26.1%) |
| Continuous Phenotype | 0 (0%) | 16 (21.3%) | 27 (40.3%) | 2 (12.5%) | 45 (27.3%) |
| Delayed Onset Symptoms Phenotype | 0 (0%) | 3 (4.0%) | 3 (4.5%) | 0 (0%) | 6 (3.6%) |

CNVS, chronic nausea and vomiting syndrome; FD, functional dyspepsia.

**Supplemental Appendix F**

**Table S4:** List of ongoing body surface gastric mapping studies (excludes completed and published studies)

| **Clinical Trial Registration Number** | **Title** | **Objective** |
| --- | --- | --- |
| NCT06854120 | Prokinetics and Body Surface Gastric Mapping in Dyspeptic Patients: Baseline and Treatment Effects | To understand how prokinetic medications affect gastric function and identify baseline BSGM factors that could predict responses to treatment. |
| NCT06381349 | Predicting Outcomes of GPOEM Using Gastric Electrical Mapping (GPOEM-GEMS) | To determine the clinical utility of Gastric Alimetry in predicting gastric per oral endoscopic pyloromyotomy treatment outcomes. |
| NCT06493032 | The Use of Body Surface Gastric Mapping in Assessing the Clinical Success of G-POEM | To investigate whether or not Body Surface Gastric Mapping can determine which patients with gastroparesis would best benefit from a GPOEM procedure. |
| NCT06941545 | High Resolution Gastric Mapping and Gastroduodenal Manometry | To identify disturbances and characterize phenotypes in patients with functional dyspepsia, and to assess the correlations between symptoms (during the manometry and in daily life), gastric emptying, electrical activity (BSGM), and pressure activity (manometry). |
| NCT05719168 | Gastric Neuromuscular Function in GERD | To assess gastric neuromuscular function in healthy controls and patients with medical refractory gastroesophageal reflux disease (GERD). |
| NCT04661215 | Pyloric Sphincter Abnormalities in Patients With Gastroparesis Symptoms (PSAGS) | To determine if there are pyloric sphincter abnormalities in patients with gastroparesis symptoms and determine how prevalent these abnormalities are using tests to assess the pyloric sphincter - endoluminal functional luminal imaging probe (Endoflip™), water load satiety testing (WLST), and high-resolution cutaneous electrogastrography (HR-EGG) using Gastric Alimetry™ System. |
| NCT06846359 | Treatment of Upper Gastrointestinal Symptoms Using Hypnotherapy Sessions | To evaluate if hypnotherapy delivered digitally will help your GI symptoms (includes change in AGBW score as a secondary outcome). |
| NCT05789511 | HR-EGG in Medically Refractory Gastroparesis | A: To assess the presence of gastric dysrhythmias in a lung transplantation population as compared to alternative etiologies of gastroparesis.  B: To assess if presence or absence of gastric dysrhythmias is predictive of response or need of GPOEM.  C: To assess alterations in gastric dysrhythmias following pyloric interventions including GPOEM. |

**Supplemental Appendix G**

This appendix summarizes the personnel, time, and interpretive resources involved in delivering a standardized body surface gastric mapping (BSGM) test as described in the Auckland Classification, compiled from the literature review and multi-centre clinical experience.

Test Workflow

| **Activity** | **Personnel** | **Time** |
| --- | --- | --- |
| **Pre-Service** | | |
| Skin preparation, array placement (8×8 pregelled Ag/AgCl patch), impedance verification, device and app initialization | Technician / RN | ~20 min |
| Review patient history and indication; confirm medication holds (≥24 h for prokinetics, opioids, antiemetics) | Physician | 5–10 min |
| **Intra-Service** | | |
| Fasting baseline recording | Automated; periodic staff check-ins | 30 min |
| Standardized meal provision (~482 kcal) | Clinical staff | ~10 min |
| Post-prandial recording with app-based symptom logging (minimum every 15 min) | Automated; periodic staff check-ins | 4 h |
| **Post-Service** | | |
| Electrode removal, data upload to cloud platform | Technician / RN | ~10 min |
| Physician interpretation: spectral analysis), symptom profiling, phenotype classification per Auckland Classification | Physician | ~35 min |
| Review results with patient; report generation | Physician | ~15 min |

Interpretation Requirements

Physician interpretation follows the four-section framework (Test Quality, Spectral Analysis, Symptoms, Conclusions) and phenotype classification described in the Auckland Classification.

| **Domain** | **Description** |
| --- | --- |
| **Technical skill** | Requires familiarity with spectral analysis of gastric electrophysiology, BMI-adjusted amplitude interpretation, and artifact recognition. |
| **Mental effort and judgment** | Integration of spectral metrics (PGF, GA-RI, BMI-adjusted amplitude, ff-AR), assessment of abnormalities, evaluation of symptom dimensions for meal-responsiveness and amplitude correlation, and synthesis into mechanism-based phenotype classification. |
| **Clinical integration** | Results require correlation with patient history, medications, prior gastric emptying testing, and comorbidities. Phenotype assignment informs mechanism-targeted therapeutic recommendations. |

**References**

1. O’Grady G, Varghese C, Schamberg G, Calder S, Du P, Xu W, et al. Principles and clinical methods of body surface gastric mapping: Technical review. Neurogastroenterol Motil. 2023 Mar 29;35(10):e14556.

2. Gharibans AA, Hayes TCL, Carson DA, Calder S, Varghese C, Du P, et al. A novel scalable electrode array and system for non-invasively assessing gastric function using flexible electronics. Neurogastroenterol Motil. 2022 June 14;35(2):e14418.

3. Huang IH, Calder S, Gharibans AA, Schamberg G, Varghese C, Andrews CN, et al. Meal effects on gastric bioelectrical activity utilizing body surface gastric mapping in healthy subjects. Neurogastroenterol Motil. 2024 Aug;36(8):e14823.

4. Calder S, Schamberg G, Varghese C, Waite S, Sebaratnam G, Woodhead JST, et al. An automated artifact detection and rejection system for body surface gastric mapping. Neurogastroenterol Motil. 2022 June 14;34(11):e14421.

5. Schamberg G, Varghese C, Calder S, Waite S, Erickson J, O’Grady G, et al. Revised spectral metrics for body surface measurements of gastric electrophysiology. Neurogastroenterol Motil. 2023 Mar;35(3):e14491.

6. Varghese C, Schamberg G, Calder S, Waite S, Carson D, Foong D, et al. Normative Values for Body Surface Gastric Mapping Evaluations of Gastric Motility Using Gastric Alimetry: Spectral Analysis. Am J Gastroenterol. 2022 Dec 20;118(6):1047–57.

7. Lim AH, Varghese C, Sebaratnam GH, Schamberg G, Calder S, Gharibans AA, et al. Effect of menstrual cycle and menopause on human gastric electrophysiology. Am J Physiol Gastrointest Liver Physiol. 2024 July 1;327(1):G47–56.

8. Lim AH, Varghese C, Sebaratnam G, Schamberg G, Calder S, Gharibans A, et al. Nausea and gastric myoelectrical activity are influenced by hormonal contraception in chronic gastroduodenal disorders. Clin Transl Gastroenterol. 2025 Sept 1;16(9):e00880.

9. Carson DA, Robertson S, Wang THH, Varghese C, Gharibans AA, Windsor JA, et al. The impact and clinical implications of gastric surgery on the gastric conduction system. Foregut (Thousand Oaks). 2023 Mar;3(1):29–44.

10. Wang THH, Tokhi A, Gharibans A, Evennett N, Beban G, Schamberg G, et al. Non-invasive thoracoabdominal mapping of postoesophagectomy conduit function. BJS Open [Internet]. 2023 May 5;7(3). Available from: http://dx.doi.org/10.1093/bjsopen/zrad036

11. Wang THH, Du P, Angeli TR, Paskaranandavadivel N, Erickson JC, Abell TL, et al. Relationships between gastric slow wave frequency, velocity, and extracellular amplitude studied by a joint experimental-theoretical approach. Neurogastroenterol Motil [Internet]. 2018 Jan;30(1). Available from: http://dx.doi.org/10.1111/nmo.13152

12. O’Grady G, Gharibans AA, Du P, Huizinga JD. The gastric conduction system in health and disease: a translational review. Am J Physiol Gastrointest Liver Physiol. 2021 Sept 22;321(5):G527–42.

13. Du P, Maharjan A, Calder S, Schultz M, Schamberg G, Gharibans A, et al. Transcutaneous Auricular Vagus Nerve Stimulation Normalizes Induced Gastric Myoelectrical Dysrhythmias in Controls Assessed by Body-Surface Gastric Mapping. Neuromodulation. 2023 Mar 29;27(2):333–42.

14. Chan CHA, Aghababaie Z, Paskaranandavadivel N, Avci R, Cheng LK, Angeli-Gordon TR. Localized gastric distension disrupts slow-wave entrainment leading to temporary ectopic propagation: a high-resolution electrical mapping study. Am J Physiol Gastrointest Liver Physiol. 2021 Oct 6;321(6):G656–67.

15. Hasler WL, Kim MS, Chey WD, Stevenson V, Stein B, Owyang C. Central cholinergic and alpha-adrenergic mediation of gastric slow wave dysrhythmias evoked during motion sickness. Am J Physiol. 1995 Apr;268(4 Pt 1):G539-47.

16. Abell TL, Malagelada JR. Glucagon-evoked gastric dysrhythmias in humans shown by an improved electrogastrographic technique. Gastroenterology. 1985 June;88(6):1932–40.

17. Gharibans AA, Hayes TCL, Carson DA, Calder S, Varghese C, Du P, et al. A novel scalable electrode array and system for non‐invasively assessing gastric function using flexible electronics. Neurogastroenterology & Motility. 2023;35(2):e14418.

18. Gharibans AA, Calder S, Varghese C, Waite S, Schamberg G, Daker C, et al. Gastric dysfunction in patients with chronic nausea and vomiting syndromes defined by a noninvasive gastric mapping device. Sci Transl Med. 2022 Sept 21;14(663):eabq3544.

19. Nederkoorn C, Smulders FT, Jansen A. Cephalic phase responses, craving and food intake in normal subjects. Appetite. 2000 Aug;35(1):45–55.

20. Gharibans AA, Smarr BL, Kunkel DC, Kriegsfeld LJ, Mousa HM, Coleman TP. Artifact rejection methodology enables continuous, noninvasive measurement of gastric myoelectric activity in ambulatory subjects. Sci Rep. 2018;8(1):5019.

21. Varghese C, Huang IH, Schamberg G, Calder S, Andrews CN, O’Grady G, et al. Distinct subgroups in gastroparesis defined by simultaneous body surface gastric mapping and gastric emptying breath testing. Neurogastroenterol Motil. 2025 July 28;e70124.

22. Varghese C, Schamberg G, Uren E, Calder S, Law M, Foong D, et al. A standardized classification scheme for gastroduodenal disorder evaluation using the Gastric Alimetry system: Prospective cohort study. Gastro Hep Adv. 2025;4(1):100547.

23. Ayubi H, Varghese C, Tanne M, Gulati S, Patel M, Haji A, et al. Predicting response to gastric per oral endoscopic myotomy: Initial results from a tertiary centre using integrated assessment with body surface gastric mapping. Gastrointest Endosc. 2025 May;101(5):S657.

24. Humphrey G, Pearlstein H, Xu B, O’Grady G, Gharibans A, Mousa H. Body surface gastric mapping facilitates the management of pediatric disorders of gut-brain interaction. Therap Adv Gastroenterol. 2025 Nov 1;18(17562848251384224):17562848251384224.

25. Sadaka C, Xu B, Benitez AJ, Orians CM, Bowerman C, Dourlain J, et al. Neuropathic gastroduodenal disorders can be diagnosed by non-invasive body surface gastric mapping: A comparison with antroduodenal manometry. Neurogastroenterol Motil. 2025 June 2;e70087.

26. Gharibans AA, Huang IH, Schamberg G, Dachs N, Tack J, O’Grady G, et al. Novel digital symptom scores for use in gastroduodenal disorder testing align with Rome IV criteria. Gastro Hep Adv. 2026;5(1):100815.

27. Ayubi H, Varghese C, Tanne M, Schamberg G, Gulati S, Patel M, et al. Body surface gastric mapping parameters are associated with response to gastric peroral endoscopic myotomy for gastroparesis: pilot study [Internet]. medRxiv. 2025 [cited 2025 Oct 9]. p. 2025.09.18.25336055. Available from: https://www.medrxiv.org/content/10.1101/2025.09.18.25336055v1.abstract

28. Wang THH, Varghese C, Calder S, Gharibans A, Schamberg G, Bartlett A, et al. Long-term evaluation of gastric electrophysiology, symptoms and quality of life after pancreaticoduodenectomy. HPB (Oxford). 2025 Dec;27(12):1535–42.

29. Varghese C, O’Grady G. Non-invasive biomarkers of gastric function from body surface gastric mapping and their role in chronic gastroduodenal disorders. Expert Rev Gastroenterol Hepatol. 2025 Aug 21;(17474124.2025.2549376):1–10.

30. Shaaban H, Varghese C, Schamberg G, Wallace I, Law M, Poonawala N, et al. Liquid nutrient drink testing induces gastric myoelectrical abnormalities that correlate with gastroduodenal symptoms. Am J Physiol Gastrointest Liver Physiol. 2025 Sept 1;329(3):G363–70.

31. Law M, Schamberg G, Varghese C, Wu B, Daker C, Pickering I, et al. Psychometric profiling of patients with chronic gastroduodenal symptoms using body surface gastric mapping phenotypes. medRxiv [Internet]. 2025 July 29; Available from: https://www.medrxiv.org/content/10.1101/2025.07.28.25332341v1.full

32. Coutinho S, Varghese C, Buenz E, Calder S, Gharibans A, O’Grady G, et al. Glucagon-like peptide-1 agonist liraglutide induces temporary impairment to gastric electrical activity in healthy volunteers. Clin Gastroenterol Hepatol [Internet]. 2025 July 29 [cited 2025 Aug 13]; Available from: http://dx.doi.org/10.1016/j.cgh.2025.07.027

33. Xu W, Simmonds S, Foong D, Bhat S, Varghese C, Andrews CN, et al. Association between gastric rhythm and gastroesophageal reflux defined by simultaneous body surface gastric mapping and 24-h pH testing. Am J Physiol Gastrointest Liver Physiol. 2026 Jan 1;330(1):G62–71.

34. Varghese C, Zhou W, Gharibans A, Schamberg G, Van Hove S, O’Grady G, et al. Autonomic dysfunction in gastroduodenal disorders evaluated through multimodal non-invasive physiological testing [Internet]. Am. J. Gastroenterol. 2025. Available from: http://dx.doi.org/10.1101/2025.06.29.25330513

35. Law M, Humphrey G, Pickering I, Schamberg G, Varghese C, Du P, et al. Validation of the youth version of the Alimetry® Gut-Brain Wellbeing Survey: A mental health scale for young people with chronic gastroduodenal symptoms [Internet]. medRxiv. 2025. Available from: http://dx.doi.org/10.1101/2025.06.05.25329082

36. Humphrey G, Keane C, Schamberg G, Benitez A, Calder S, Binghong X, et al. Body Surface Gastric Mapping delineates specific patient phenotypes in adolescents with functional dyspepsia and gastroparesis. Neurogastroenterol Motil. 2025 Mar 19;e70018.

37. Varghese C, Gharibans AA, Foong D, Schamberg G, Calder S, Ho V, et al. Relationship between intragastric meal distribution, gastric emptying, and gastric neuromuscular dysfunction in chronic gastroduodenal disorders. Neurogastroenterol Motil. 2025 Sept 22;(e70170):e70170.

38. Simmonds S, Foong D, Schamberg G, Johnston G, Ho V, Hobson A, et al. Vomiting during body surface gastric mapping testing [Internet]. medRxiv. 2025. Available from: http://dx.doi.org/10.1101/2025.10.08.25337635

39. Wang THH, Varghese C, Calder S, Gharibans AA, Evennett N, Beban G, et al. Assessment of gastric remnant activity, symptoms, and quality of life following gastric bypass. Obes Surg. 2024 Dec;34(12):4490–8.

40. Law M, Bartlett E, Sebaratnam G, Pickering I, Simpson K, Keane C, et al. One more tool in the tool belt: A qualitative interview study investigating patient and clinician opinions on the integration of psychometrics into routine testing for disorders of gut-brain interaction. Cureus. 2024 Aug 18;16(8):e67155.

41. Law M, Pickering I, Humphrey G, Sebaratnam G, Schamberg G, Simpson K, et al. Development and validation of the Alimetry Gut-Brain Wellbeing Survey: a novel patient-reported mental health scale for patients with chronic gastroduodenal symptoms. Front Psychol. 2024 July 8;15(1389671):1389671.

42. Law M, Schamberg G, Gharibans A, Sebaratnam G, Foong D, Varghese C, et al. Short- and long-term reproducibility of body surface gastric mapping using the Gastric Alimetry® system. Neurogastroenterol Motil. 2024 July;36(7):e14812.

43. Huang IH, Schol J, Calder S, Gharibans AA, Van den Houte K, Verheyden A, et al. Effects of corticotropin-releasing hormone on gastric electrical activity and sensorimotor function in healthy volunteers: a double-blinded crossover study. Am J Physiol Gastrointest Liver Physiol. 2024 May 1;326(5):G622–30.

44. Humphrey G, Keane C, Gharibans A, Andrews CN, Benitez A, Mousa H, et al. Designing, developing, and validating a set of standardized pictograms to support pediatric-reported gastroduodenal symptoms. J Pediatr. 2024 Apr;267(113922):113922.

45. Xu W, Wang T, Foong D, Schamberg G, Evennett N, Beban G, et al. Characterization of gastric dysfunction after fundoplication using body surface gastric mapping. J Gastrointest Surg. 2024 Mar;28(3):236–45.

46. Wang WJ, Foong D, Calder S, Schamberg G, Varghese C, Tack J, et al. Gastric Alimetry expands patient phenotyping in gastroduodenal disorders compared with gastric emptying scintigraphy. Am J Gastroenterol. 2024 Feb 1;119(2):331–41.

47. Foong D, Calder S, Varghese C, Schamberg G, Xu W, Daker C, et al. Gastric Alimetry® Test Interpretation in Gastroduodenal Disorders: Review and Recommendations. J Clin Med Res [Internet]. 2023 Oct 10;12(20). Available from: http://dx.doi.org/10.3390/jcm12206436

48. Schamberg G, Calder S, Varghese C, Xu W, Wang WJ, Ho V, et al. Comparison of Gastric Alimetry® body surface gastric mapping versus electrogastrography spectral analysis. Sci Rep. 2023 Sept 11;13(1):14987.

49. Xu W, Gharibans AA, Calder S, Schamberg G, Walters A, Jang J, et al. Defining and phenotyping gastric abnormalities in long-term type 1 diabetes using a novel body surface gastric mapping device. Gastro Hep Adv. 2023 Aug 18;2(8):1120–32.

50. O’Grady G, Carbone F, Tack J. Gastric sensorimotor function and its clinical measurement. Neurogastroenterol Motil. 2022 Dec;34(12):e14489.

51. Calder S, Cheng LK, Andrews CN, Paskaranandavadivel N, Waite S, Alighaleh S, et al. Validation of noninvasive body-surface gastric mapping for detecting gastric slow-wave spatiotemporal features by simultaneous serosal mapping in porcine. Am J Physiol Gastrointest Liver Physiol. 2022 Aug 2;323(4):G295–305.

52. Sebaratnam G, Karulkar N, Calder S, Woodhead JST, Keane C, Carson DA, et al. Standardized system and App for continuous patient symptom logging in gastroduodenal disorders: Design, implementation, and validation. Neurogastroenterology & Motility. 2022 Aug 1;34(8):e14331.

53. Carson DA, O’Grady G, Du P, Gharibans AA, Andrews CN. Body surface mapping of the stomach: New directions for clinically evaluating gastric electrical activity. Neurogastroenterol Motil. 2021 Mar;33(3):e14048.

54. Somarajan S, Muszynski ND, Olson JD, Comstock A, Russell AC, Walker LS, et al. The effect of chronic nausea on gastric slow wave spatiotemporal dynamics in children. Neurogastroenterol Motil. 2021 May;33(5):e14035.

55. Gharibans AA, Coleman TP, Mousa H, Kunkel DC. Spatial patterns from high-resolution electrogastrography correlate with severity of symptoms in patients with functional dyspepsia and gastroparesis. Clin Gastroenterol Hepatol. 2019 Dec;17(13):2668–77.

56. O’Grady G, Angeli TR, Paskaranandavadivel N, Erickson JC, Wells CI, Gharibans AA, et al. Methods for High-Resolution Electrical Mapping in the Gastrointestinal Tract. IEEE Rev Biomed Eng. 2019;12:287–302.

57. Gharibans AA, Kim S, Kunkel DC, Coleman TP. High-resolution electrogastrogram: a novel, noninvasive method for determining gastric slow-wave direction and speed. IEEE Transactions on Biomedical Engineering. 2016;64(4):807–15.

58. Abell TL, Camilleri M, Donohoe K, Hasler WL, Lin HC, Maurer AH, et al. Consensus Recommendations for Gastric Emptying Scintigraphy: A Joint Report of the American Neurogastroenterology and Motility Society and the Society of Nuclear Medicine. J Nucl Med Technol. 2008 Mar 1;36(1):44–54.

59. Karamanolis G, Caenepeel P, Arts J, Tack J. Determinants of symptom pattern in idiopathic severely delayed gastric emptying: gastric emptying rate or proximal stomach dysfunction? Gut. 2007 Jan;56(1):29–36.

60. Stanghellini V, Talley NJ, Chan F, Hasler WL, Malagelada J, Suzuki H, et al. Rome IV - gastroduodenal disorders. Gastroenterology. 2016 Feb 15;150(6):1380–92.
